# Intrinsic structural dynamics dictate enzymatic activity and inhibition

**DOI:** 10.1073/pnas.2310910120

**Published:** 2023-10-02

**Authors:** Vaibhav Kumar Shukla, Lucas Siemons, D. Flemming Hansen

**Affiliations:** ^a^Division of Biosciences, Department of Structural and Molecular Biology, University College London, London WC1E 6BT, United Kingdom

**Keywords:** enzymes, protein dynamics, histone deacetylase, methyl-TROSY NMR, CPMG relaxation dispersion

## Abstract

Enzymes are known to sample various conformations, many of which are critical for their biological function. However, structural characterizations of enzymes predominantly focus on the most populated conformation. As a result, single-point mutations often produce structures that are similar or essentially identical to those of the wild-type enzyme despite large changes in enzymatic activity. Here, we show for mutants of a histone deacetylase enzyme (HDAC8) that reduced enzymatic activities, reduced inhibitor affinities, and reduced residence times are all captured by the rate constants between intrinsically sampled conformations that, in turn, can be obtained independently by solution NMR spectroscopy. Thus, for the HDAC8 enzyme, the dynamic sampling of conformations dictates both enzymatic activity and inhibitor potency. Our analysis also dissects the functional role of the conformations sampled, where specific conformations distinct from those in available structures are responsible for substrate and inhibitor binding, catalysis, and product dissociation. Precise structures alone often do not adequately explain the effect of missense mutations on enzymatic activity and drug potency. Our findings not only assign functional roles to several conformational states of HDAC8 but they also underscore the paramount role of dynamics, which will have general implications for characterizing missense mutations and designing inhibitors.

## Significance

Enzymes sample different conformations on the millisecond timescale, conformations that are often imperative for their biological function. For a histone deacetylase enzyme (HDAC8), and mutants therefore, we show that four global kinetic parameters and a model of the intrinsic dynamics accurately describe the enzymatic activity, inhibitor affinity, and inhibitor residence time. Our findings suggest that changes in enzymatic activity and inhibitor potency are all captured in the differences in the intrinsic dynamics. Our study not only enhances our understanding of enzymatic regulations and dissects the effect of missense mutants, but since enzymes are targets in most drug discovery programs, our study also has the potential to improve drug developments and rational enzyme designs.

Couplings between enzymatic activity and intrinsic dynamics have recently been shown experimentally for several enzymes (1–3), and theoretical molecular dynamics (MD) simulations have provided additional insights into the functional dynamics of several enzymes (4–8). NMR is an experimental technique, highly complementary to MD simulations, which has evolved into an important tool to experimentally characterize the functional dynamics of enzymes (1, 9–12). Within biomolecular NMR, methyl transverse relaxation optimized spectroscopy (TROSY) (1, 13–17) techniques now provide insights on large proteins and enzymes, and methods such as Carr-Purcell-Meiboom-Gill (CPMG) relaxation dispersion are now available to characterize millisecond conformational samplings of macromolecules in solution (2, 12, 18–22).

Previous work has shown that structural millisecond dynamics of kinases, chaperones (23–25), reductases (26), and hydrolases (3) are important for biological function and that stabilizations of alternate conformations can lead to diseases (12). The class I histone deacetylase HDAC8, a hydrolase, is an enzyme that has recently garnered substantial attention due to its notable role in several cancer subtypes (27, 28), as well as in Cornelia de Lange syndrome (CdLS) (29–33). Deacetylation is a widespread phenomenon among eukaryotes to regulate gene expression (34) and it is therefore unsurprising that changes to deacetylation levels as a result of altered expression or aberrant regulation of HDACs lead to several diseases ranging from neurodegenerative disorders to cancers (35–37), with HDAC inhibitors highlighted as an important category of anticancer drugs. Despite HDAC8 being the human HDAC for which high-resolution structures first became available, now with more than 50 structures of HDAC8 and its mutants available in complex with substrates or inhibitors (31, 32, 38–41), a structure of the free HDAC8 (unbound active state) is not yet available. As for many other enzymes, key insight has been gained into the catalytic mechanism of HDAC8 from the available structures and biochemical data; however, several inactive mutants of HDAC8 produce crystal structures that are "essentially identical" to the active wild-type (42). This poses a substantial challenge for rationalizing the effect of missense mutations and suggests that the effect of some missense mutations on activity is beyond a perturbation to the major crystallized conformation (3, 43). Moreover, the fact that crucial residues implicated in catalysis are associated with loop regions (4) clearly points to the importance of dynamics for function and regulation. Thus, the ability to characterize the coupling between intrinsic structural dynamics and enzymatic activity, not only for HDACs but also for enzymes more broadly, is crucial for understanding enzymatic regulation and the effects of missense mutants at a level where the insights can aid the development of improved treatments.

## Results

### Distinct Intrinsic Millisecond Dynamics of HDAC8 in Three Regions.

**Fig. 1.**
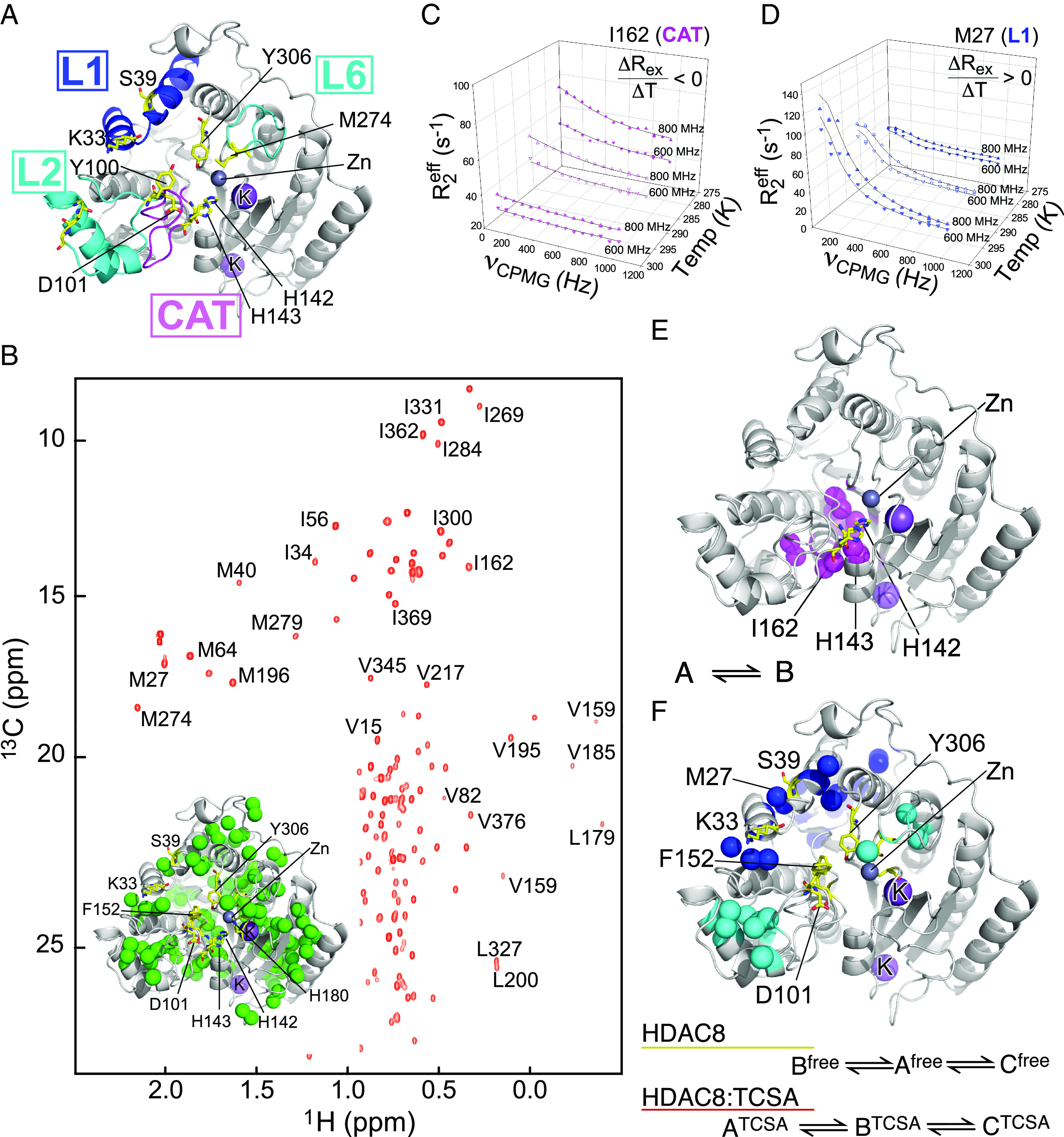
Millisecond dynamics in the free and bound states of HDAC8. (*A*) Crystal structure of HDAC8 (38) highlighting regions that show millisecond dynamics. Residues crucial for deacetylation activity and regulation are highlighted and shown in yellow sticks. (*B*) Methyl-TROSY NMR spectrum of HDAC8 acquired on a Ile, Leu, Val, Met (ILVM) methyl-labeled sample at a static magnetic field strength of 22.3 T. The chemical shift assignment for the methyl groups of 23 Ile, 26 Val, 32 Leu, and 10 Met were obtained as described in the Methods section. The inset shows all assigned methyl groups of ILVM as green spheres on a cartoon representation of HDAC8 [PDB ID:1T64] (38). (*C* and *D*) Three-dimensional plots of MQ-CPMG profiles for I162 (*C*), a representative residue of the catalytic region, CAT, and M27 (*D*), which is a representative residue of the loop regions. $\begin{array}{ccccc} R_{ex} & = & R_{2}^{\mathrm{eff}}\left(\nu_{\mathrm{CPMG}}=0\right) & - & R_{2}^{\mathrm{eff}}\left(\nu_{\mathrm{CPMG}}\to \infty \right) \end{array}$ . In (*C* and *D*), the MQ-CPMG data of HDAC8 bound to the inhibitor (HDAC8:TCSA) were acquired at magnetic field strengths of 18.8 T and 14.1 T and at temperatures of 298 K, 288 K, and 278 K. (*E*) The methyl groups of HDAC8 that show two conformationally exchanging states (CAT region). (*F*) Methyl groups of HDAC8 that show three conformationally exchanging states (loop regions). The exchanging states, denoted A, B, and C, are ordered based on their populations.

MD simulations have previously shown the presence of several stable conformations of HDAC8 as well as a coupling between the aromatic residues near the active site and the surrounding loop regions (4, 44). The conformational dynamics in the vicinity of one of these loops, loop 1 (L1) (Fig. 1*A*), was previously probed experimentally using methyl-TROSY multiquantum CPMG (MQ-CPMG) (17) experiments of isoleucine residues. It was revealed that the population of a sparsely populated conformation of HDAC8 correlates with enzymatic activity (3).

In the current study, a near-complete chemical shift assignment of the isoleucine, valine, leucine, and methionine methyl groups in HDAC8 is achieved (Fig. 1*B* and *SI Appendix*, Fig. S1 and Table S1), which forms an excellent basis for a characterization of the conformational sampling of HDAC8, since the assigned residues are well dispersed in the three-dimensional structure and cover ~25% of the primary sequence. Following the chemical shift assignment, MQ-CPMG experiments were acquired at three different temperatures on free HDAC8 and on the enzyme in complex with the inhibitor, trichostatin **A** (TCSA). The TCSA inhibitor has been shown to interact with HDAC8 with a slow overall off-rate (0.1 s^−1^) (45), thus ensuring that the binding kinetics is invisible to the MQ-CPMG methods and that the MQ-CPMG dispersions observed for the HDAC8:TCSA complex originate from unimolecular and intrinsic structural dynamics as also shown previously (3). Thus, the TCSA inhibitor facilitates the generation of a homogenous complex that resembles substrate/product-bound enzyme.

The set of MQ-CPMG data obtained for free HDAC8 and for HDAC8:TCSA initially revealed at least two distinct groups of millisecond dynamics: One group of dynamics centered around the catalytic site, hereafter referred to as CAT (Fig. 1 *C* and *E* and *SI Appendix*, Fig. S2), while another group of dynamics was associated with loop 1 (L1), loop 2 (L2), and loop 6 (L6) (Fig. 1 *D* and *F* and *SI Appendix*, Fig. S2). Upon addition of TCSA and the formation of the HDAC8:TCSA complex, substantial changes in the chemical shifts were observed only for the methyl groups of the L1, L2, and L6 regions (*SI Appendix*, Fig. S3 *A* and *C*). However, all four regions, including the CAT region, showed changes in the MQ-CPMG dispersions upon binding of the inhibitor TCSA (*SI Appendix*, Fig. S3 *D* and *E*). In the catalytic region (CAT), the relaxation dispersions were quenched upon TCSA binding, while dispersion originating from the loop regions increased (*SI Appendix*, Fig. S3 *D* and *E*). Therefore, millisecond dynamics observed in the CAT region differs from the three loop regions in both free HDAC8 and HDAC8:TCSA. For methyl groups near L1 in free HDAC8, the dispersions increase with a decrease in temperature, while in HDAC8:TCSA, the dispersions decrease with a decrease in temperature. In contrast, for methyl groups in the L2 and L6 regions, the dispersions increase with a decrease in temperature for both free and TCSA-bound HDAC8. Thus, based on their different temperature dependences, methyl groups showing millisecond dynamics in the three loop regions were further grouped into two clusters, namely L1 and L2,6. Subsequent F-test analysis showed that it was not statistically significant to treat L2 and L6 separately. Overall, residues showing millisecond dynamics in HDAC8 could confidently be grouped into three regions: L1; L2,6; and CAT, as depicted in Fig. 1*A* and detailed in *SI Appendix*, Tables S2 and S3*A* and Fig. S2.

### Two Conformations Sampled in the CAT Region and Three Conformations Sampled in the L1 and L2,6 Regions.

**Fig. 2.**
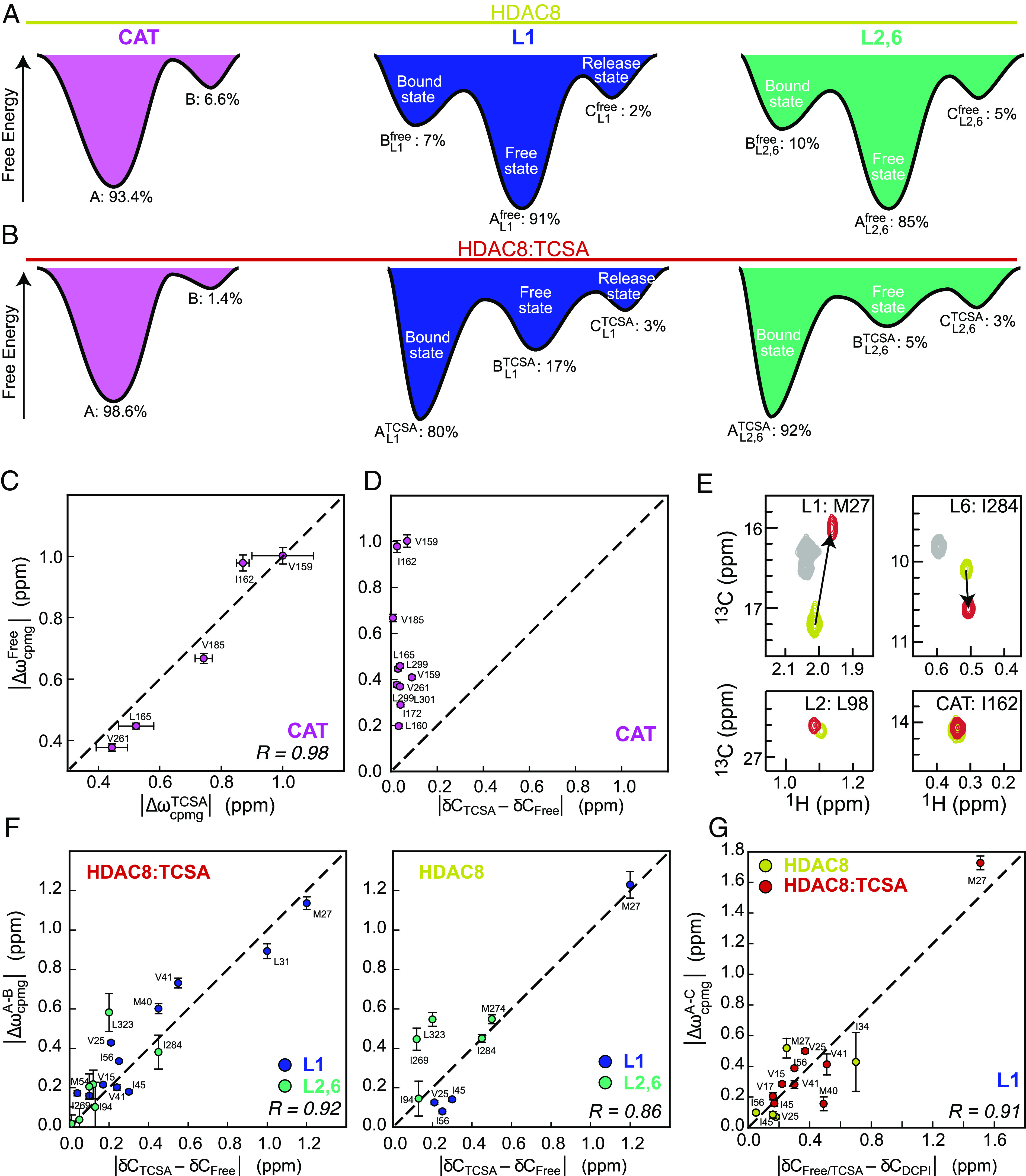
Identifying the nature of the exchanging conformations. (*A* and *B*) show schematically the Gibbs free-energy landscape derived from the MQ-CPMG data for the CAT region (*Left*), the L1 region (*Middle*), and the L2,6 region (*Right*) in free HDAC8 and HDAC8:TCSA, respectively. The nature of the conformations is annotated, where known. (*C*) Correlation between |Δω_A,B_| obtained from MQ-CPMG for residues in the CAT region for free HDAC8 (*y* axis) and HDAC8:TCSA (*x* axis). Vertical and horizontal lines represent the SD of the |Δω_A,B_| obtained in the least-squares fit (*Materials and Methods*) for free HDAC8 and HDAC8:TCSA, respectively. (*D*) Correlation between ^13^C chemical shift differences between free HDAC8 and HDAC8:TCSA (*x* axis) and |Δω_A,B_| obtained from CPMG relaxation dispersion experiments for the CAT region. (*E*) The chemical shift perturbation of representative residues of the four regions upon TCSA binding to HDAC8. (*F*) Correlation between ^13^C chemical shift differences between free and TCSA-bound HDAC8 (*x* axis), and |Δω_A,B_| obtained from CPMG relaxation dispersion experiments for $B_{L1}^{TCSA}$ , and $B_{L2,6}^{TCSA}$ state of HDAC8:TCSA (*Left*) and $B_{L1}^{free}$ and $B_{L2,6}^{free}$ of free HDAC8 (*Right*), respectively. Vertical lines represent the SD of the derived |Δω_A,B_|. (*G*) Correlation between ^13^C chemical shift differences between free/TCSA- and DCPI-bound HDAC8 (*x* axis) and |Δω_A,C_| of the $C_{L1}^{free}$ and $C_{L1}^{TCSA}$ state for the L1 region in HDAC8.

The MQ-CPMG data for the CAT region were consistent with a two-site exchange model, A ⇌ B, where the majorly populated conformation (A) is in exchange with a single minorly populated conformation (B). Along with the methyl-specific ^13^C chemical shift differences, Δω = ω_B_ – ω_A_, four global parameters were obtained to describe the unimolecular reaction near the catalytic site, namely ΔH_B_, ΔH^‡^, ΔS_B_, and ΔS^‡^, where ΔH_B_ and ΔS_B_ are the enthalpy and entropy of the sparsely populated conformation B relative to A, and ΔH^‡^ and ΔS^‡^ are the enthalpy and entropy of the transition state relative to A (17, 46) (*SI Appendix*, Fig. S4). The four global parameters can be converted to populations and microkinetic rates at a given temperature using standard formulas (46) (Fig. 2 *A* and *B* and *SI Appendix*, Table S3*B*).

Initially, an attempt was made to analyze the free HDAC8 MQ-CPMG data for the L1 and L2,6 regions with a two-site exchange model as for the CAT region; however, some residues were inconsistent with this model over the range of temperatures used. Therefore, analyses with three-site exchange models were carried out, which showed that a so-called bifurcated model, B^free^ ⇌ A^free^ ⇌ C^free^, was most appropriate for both L1 and L2,6 and significantly better than a two-site model (*P*-value < 10^−6^) (Fig. 2*A* and *SI Appendix*, Table S2). As indicated in *SI Appendix*, Table S2, the linear (A^free^ ⇌ B^free^ ⇌ C^free^) and bifurcated (B^free^ ⇌ A^free^ ⇌ C^free^) models resulted in similar reduced *χ*^2^ for the L1 region, and the chemical shifts obtained for B^free^ and C^free^ are also independent of the model. Based on overall considerations (see below), the bifurcated model was chosen, within which A^free^ is the major state, which means that the population of A^free^ is larger than the populations of B^free^ and C^free^.

Similarly, in the analysis of the inhibitor-bound HDAC8, HDAC8:TCSA, MQ-CPMG data of L1 and L2,6 regions with a two-site exchange model led to poor fits, which again improved significantly when a three-site exchange model was used (*P*-value < 10^−6^). In contrast to free HDAC8, the linear model, A^TCSA^ ⇌ B^TCSA^ ⇌ C^TCSA^, showed the lowest reduced *χ*^2^ (0.82 vs. 0.86 for L1 and 0.42 vs. 0.45 for L2,6) and was thus in better agreement with the data (*SI Appendix*, Table S2). The derived populations at 298 K are shown in Fig. 2*B*. In conclusion, for both free HDAC8 and HDAC8:TCSA, the L1 and L2,6 regions dynamically sample three conformations, one major and two sparsely populated conformations, while the CAT region samples only two conformations on the millisecond time-scale. For each region, final parameters obtained from the least-squares analyses are summarized in *SI Appendix*, Table S3, and free-energy, enthalpy, and entropy diagrams are shown in *SI Appendix*, Fig. S4. In order to assess how, if at all, the sparsely sampled conformations are relevant for enzymatic activity additional insight is necessary.

### Conformations Sampled in HDAC8 are Implicated in Substrate Association and Product Dissociation.

In both free HDAC8 and HDAC8:TCSA, the CAT region samples two conformations (Fig. 2 *A* and *B*), and a strong correlation between the obtained Δω for free HDAC8 and HDAC8:TCSA, (*R* = 0.98), is observed (Fig. 2*C*), which suggests that the major conformation, A, as well as the sparsely populated conformation, B, for the CAT region are very similar in free HDAC8 and in HDAC8:TCSA. In methyl-TROSY spectra, no significant changes in chemical shifts were observed for residues near the CAT region upon inhibitor binding, (Fig. 2 *D* and *E*). However, a decrease in the population of the minor conformation, *p*_B_, from 6.6% to 1.4% at 298K was observed upon inhibitor binding (Fig. 2 *A* and *B*), which shows that the inhibitor stabilizes the ground state of this region. Spectra recorded at different pH values (8.0, 7.5, 7.0) showed no chemical shift changes for residues in the CAT region, and it is therefore unlikely that the exchange observed is related to protonation/deprotonation of H143 or H142 near the CAT region. Candidates for the conformations sampled can be gleaned from previous long, unbiased all-atom MD simulations, where histidine H143 was found to sample two χ_1_ conformations, leading to a closed and an open conformation; the open conformation was previously proposed to be implicated in the release of the lysine product (4).

To gain insight into the conformations sampled in the L1 and L2,6 regions, the obtained chemical shift differences, |Δω_A,B_| = |ω_B_ – ω_A_| and |Δω_A,C_| = |ω_C_ – ω_A_|, were compared with chemical shift differences observed in methyl-TROSY spectra upon binding of the inhibitor TCSA and the HDAC8-specific inhibitor (R)-2-amino-3-(2,4-dichlorophenyl)-1-(1,3-dihydroisoindol-2-yl)-propan-1-one (DCPI) (47–49). The TCSA inhibitor is often considered a substrate-like inhibitor (38), whereas DCPI is known to open the acetate release channel near the L1 region (47). For both free HDAC8 and HDAC8:TCSA, there are strong correlations between |Δω_A,B_| and the chemical shift differences observed upon TCSA binding (Fig. 2*F*), and this correlation holds for both the L1 and L2,6 regions. This observation suggests that the $B_{L1}^{free}$ and $B_{L2,6}^{free}$ sparse conformations in free HDAC8 are similar to the major conformations, $A_{L1}^{TCSA}$ , $A_{L2,6}^{TCSA}$ , respectively, in HDAC8:TCSA and that $B_{L1}^{TCSA}$ and $B_{L2,6}^{TCSA}$ in HDAC8:TCSA are similar to the major conformations, $A_{L1}^{free}$ and $A_{L2,6}^{free}$ , of free HDAC8. We hereafter denote $A_{L1}^{free}$ , $A_{L2,6}^{free}$ , $B_{L1}^{TCSA}$ , and $B_{L2,6}^{TCSA}$ free-like conformations and $B_{L1}^{free}$ , $B_{L2,6}^{free}$ , $A_{L1}^{TCSA}$ , and $A_{L2,6}^{TCSA}$ for bound-like conformations. Moreover, the correlations observed in Fig. 2*F* combined with the significant assignment (*SI Appendix*, Table S2) of the linear model to HDAC8:TCSA substantiate that the exchange models for L1 and L2,6 in free HDAC8 are bifurcated.

To characterize the $C_{L1}^{free}$   , $C_{L2,6}^{free}$   , $C_{L1}^{TCSA}$   , and $C_{L2,6}^{TCSA}$   conformations, the |Δω_A,C_| differences obtained for free HDAC8 and for HDAC8:TCSA were compared with the changes in chemical shifts observed upon binding of the HDAC8-specific inhibitor DCPI. A strong correlation is observed for L1, (*R* = 0.91, Fig. 2*G*), which suggests that the $C_{L1}^{free}$   conformation of free HDAC8 and $C_{L1}^{TCSA}$   of HDAC8:TCSA, in the L1 region, is a conformation related to acetate product release. As a further test of the assignment of states obtained above, the plots in Fig. 2 *F* and *G* were re-produced, but with swapped *x* axes, such that |Δω_A,C_| was plotted against |δC_TCSA_ – δC_free_|, etc. This led to correlation coefficients of 0.90, 0.65, and 0.73, which are substantially lower that those shown in Fig. 2 *F* and *G*, thus substantiating the state assignment. Single-point mutations, inhibitor affinities, and enzymatic assays are used below to gain further insight into the functional role of the sampled conformations of HDAC8, including the role of $C_{L2,6}^{free}$ and $C_{L2,6}^{TCSA}$ in the L2,6 region.

### The Functional Role of the Identified Conformations.

**Fig. 3.**
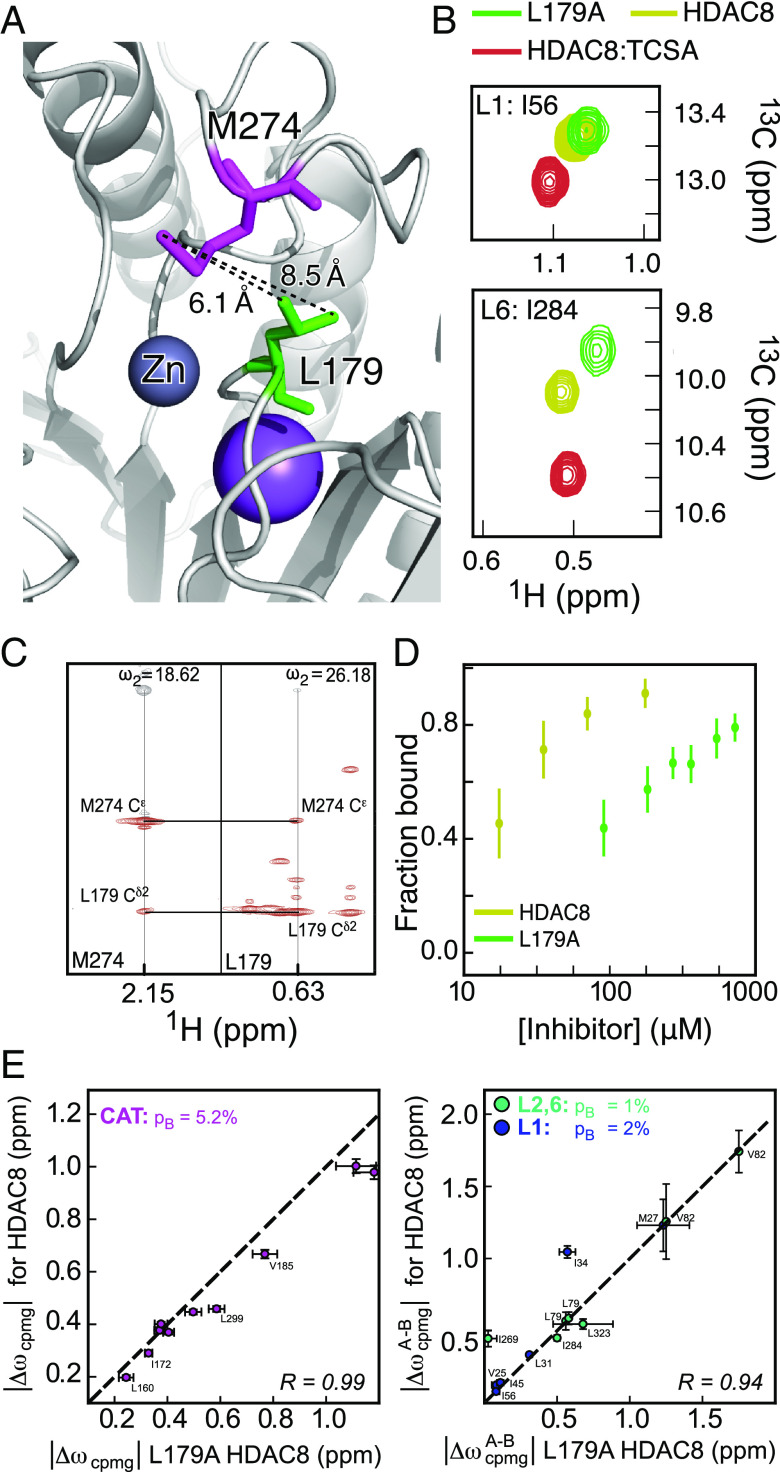
The L179A mutant alters the populations of the sampled states. (*A*) The positions of L179 and M274 in the L6 loop of HDAC8 (38), with the distance in Ångström between the two methyl groups in HDAC8. (*B*) Overlay of methyl-TROSY NMR spectra of free HDAC8 (yellow), HDAC8 with 2.5eq TCSA (red), and L179A-HDAC8 (green). Two residues are shown, I56 in the L1 loop (*Top*) and I284 in the L6 loop (*Bottom*). (*C*) 3D HMQC-NOESY strip for the methyl plane of L179 and M274 in free HDAC8, showing the NOE cross-peaks. (*D*) An NMR titration of wild-type HDAC8 and L179A-HDAC8 with the TCSA inhibitor. Each data point in the plot represents the average of the ratio of the populations of the free and bound state for five residues I45, I56, I269, I284, and I331. A SD (vertical bar) for each titration point was calculated from the fractions of the five residues. (*E*) Correlation between |Δω_A,B_| obtained for the CAT region from MQ-CPMG experiments on free HDAC8 and L179A-HDAC8 (*Left*) and correlation between |Δω_A,B_| from MQ-CPMG experiments for the B^free^ conformation of L1 and L2,6 regions in free HDAC8 and in L179A-HDAC8 (*Right*).

To gain insight into role of each of the sampled conformations for binding, catalysis/hydrolysis, and release of the acetate and lysine products, we sought to stabilize one of the sparsely populated conformations by site-directed mutagenesis (1). In the crystal structure of inhibitor-bound HDAC8, the methyl groups of L179 are in close proximity to the methyl group of M274 in L6 (38) (Fig. 3*A*). To substantiate this interaction, in a 3D ^13^C HMQC-NOESY-HMQC (50) experiment of free HDAC8, an NOE was observed between ^1^H^ε^ of M274 and ^1^H^δ2^ of L179 (Fig. 3*C*), showing that these sites are spatially within *ca.* 5 Å in free HDAC8 and thus indicating that the conformation of the L6 loop is stabilized by hydrophobic interactions between the side chains of L179 and M274. The single-point mutant, L179A, was therefore generated to perturb the population of the sampled conformations and further investigate the role of the conformational sampling of the L2,6 region for function.

Upon mutating leucine 179 to alanine (L179A), significant chemical shift perturbations (CSPs) were observed at or near the L6 region, notably at I269, I284, M274, M279, I231, and I362, whereas only small CSPs were observed for methyl resonances in the L1 region (Fig. 3*B* and *SI Appendix*, Fig. S5*A*). For most of the methyl resonances that show a significant CSP, the cross-peak moves away from both the free HDAC8 peak and the HDAC8:TCSA peaks, suggesting a destabilization of the bound and free conformations. To further corroborate this hypothesis, MQ-CPMG experiments for the L179A mutant were recorded as above for free HDAC8 at different temperatures and multiple static magnetic fields.

Linear correlations were observed between |Δω| derived for wild-type HDAC8 and L179A-HDAC8 (Fig. 3*E* and *SI Appendix*, Fig. S5*B*), suggesting that L179A-HDAC8 samples conformations that are similar to wild-type HDAC8, however, with different populations and exchange rates. For example, the population of $B_{L2,6}^{free}$ in the L2,6 region (similar to the HDAC8:TCSA ground state) was found to decrease by nearly an order of magnitude at 298 K (*SI Appendix*, Fig. S5*D*), which likely is a result of the loss of the hydrophobic interaction between L179 and M274. In contrast, the population of the $C_{L2,6}^{free}$ conformations in the L2,6 region was found to increase from 5 to 25% (*SI Appendix*, Fig. S5*E*). Small changes in the population and exchange rate were also observed for the CAT region, (*SI Appendix*, Fig. S5*F*). In an enzyme kinetics assay, the L179A mutation had a reduced deacetylase activity (*k*_cat_*/*K_M_) of 7 ± 2% compared to wild-type HDAC8. Furthermore, titration of L179A-HDAC8 with TCSA (Fig. 3*D*), showed a decrease in binding affinity, with K_d_ changing from 5 ± 2 µM in wild-type to 110 ± 11 µM in L179A-HDAC8. Since TCSA acts as a competitive inhibitor, a change in K_d_ reasonably reflects the change in K_M_. The 10-fold increase observed in K_d_ corresponds approximately to the change in the population of the $B_{L2,6}^{free}$ (bound states) from 10% in wild-type HDAC8 to 1% in L179A-HDAC8. The conformational sampling of the L2,6 region is thus highly correlated with substrate binding.

Several mutations in the L1 region were shown previously to reduce the enzymatic activity of HDAC8 (3), specifically the double mutant S39E,M40A, which led to an activity of 19 ± 4% compared to wild-type. No significant difference in the TCSA affinity of S39E,M40A-HDAC8 and wild-type HDAC8 was observed, which is consistent with the previously reported very small change in K_M_ for the S39E mutant (51). The reduced activities of S39E,M40A-HDAC8 and S39E-HDAC8 are thus a result of changes in *k*_cat_, originating from a change to the release of the product acetate. This suggests that the L1 region acts as a site for release of acetate, in agreement with Fig. 2*G*.

### A Model for Enzymatic Activity and Inhibitor Interaction Underpinned by the Internal Dynamics.

**Fig. 4.**
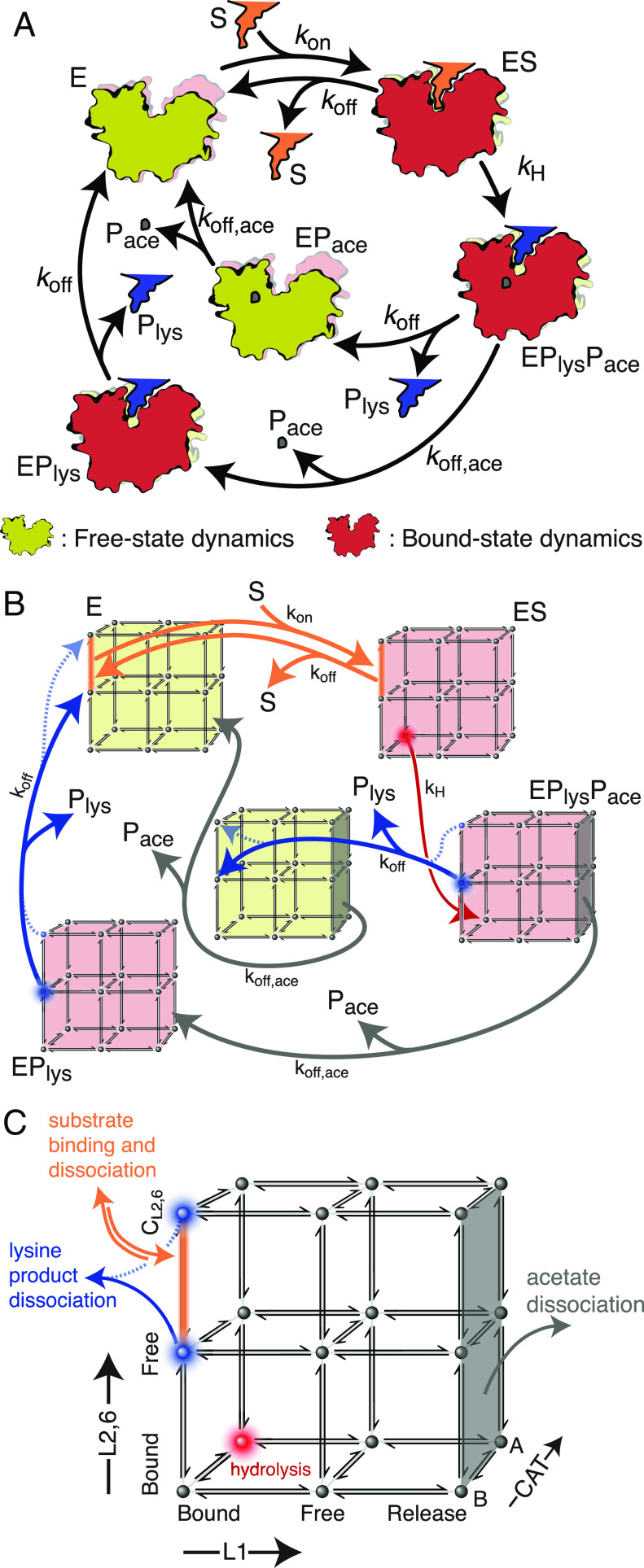
HDAC8 exploits an induce-fit-like mechanism. (*A*) Schematic representation of the catalytic cycle of HDAC8 with microkinetic rate constants obtained in the analysis. E is the free HDAC8 enzyme, ES is the substrate-bound enzyme, EP_lys_P_ace_ is the enzyme bound to the two products, EP_ace_ is the enzyme bound only to the acetate product, and EP_lys_ is the enzyme bound only to the lysine product. (*B*) Best-fit kinetic model (solid lines; model 1), where all the internal rate constants were obtained from the analysis of the MQ-CPMG-relaxation dispersion data. The specific model shown is the one with the lowest *χ*^2^ (see text). The optimized parameters are *k*_on_ = 4.0 ± 1.3 μM^−1^ s^−1^*k*_off_ = 5.1 × 10^5^ ± 1.7 × 10^5^ s^−1^, and *k*_H_(H_2_O) = 2.04 ± 0.16 s^−1^. The second-best model (model 2), *SI Appendix*, Fig. S7, with a cross-validation *P*-value of 0.80, is shown with dashed lines. (*C*) Summary of the 18 possible internal conformational states of HDAC8 based on the CPMG relaxation dispersions, showing which states are involved in hydrolysis, substrate binding, and product release. Each of the unimolecular rate constants between the 18 states is taken directly from the analysis of the MQ-CPMG data for wild-type HDAC8 and L179A-HDAC8. The second-best model (model 2) is shown with dashed lines.

The overall catalytic cycle of HDAC8 (Fig. 4*A*) includes the enzymatic species, E, ES, EP_lys_P_ace_, EP_ace_, and EP_lys_ which each exhibit intrinsic dynamics. Here, E is the free HDAC8 enzyme, ES is HDAC8 bound to substrate, EP_lys_P_ace_ is HDAC8 bound to both the lysine and acetate product, EP_ace_ is HDAC8 bound only to the acetate product, and EP_lys_ is HDAC8 bound only to the lysine product. The hydrolysis step is ES→EP_lys_P_ace_.

Before assessing the functional role of each of the sampled states, it is of interest to gain further insight into potential rate-limiting steps. The microkinetic hydrolysis of HDAC8 has previously been suggested to be rate-limited by the nucleophilic attack of H_2_O (52), which in turn comprises the break of a H-O bond in a water molecule. As such, if the hydrolysis step is rate-limiting, then one would expect to observe a large solvent deuterium kinetic isotope effect of about a factor of 8.8 (*SI Appendix, Supplementary Methods*). To investigate this, enzymatic assays were carried out at different ratios of H_2_O and D_2_O (*SI Appendix*, Fig. S6*A*), where it is seen that *k*_cat_/K_M_(H_2_O) = (6.2 ± 0.3) × *k*_cat_/K_M_(D_2_O). Subsequent MQ-CPMG experiments and inhibitor binding experiments showed that no significant differences were observed for the intrinsic dynamics nor the inhibitor dissociation constant in H_2_O and D_2_O (*SI Appendix*, Fig. S6 *B*–*F*). Therefore, the microkinetic hydrolysis is partly rate-limiting for the overall enzymatic reaction of HDAC8.

To assess the role of the intrinsic dynamics for overall catalysis, the species E and EP_ace_ were assumed to exhibit intrinsic conformational sampling similar to free HDAC8 since they have a free substrate-binding tunnel. Similarly, it is assumed that the intrinsic dynamics of ES, EP_lys_P_ace_, and EP_lys_ are similar to the intrinsic dynamics of the HDAC8:TCSA complex. Finally, it is assumed that the motions in the three regions are uncorrelated. From the results above, it is clear that the motions in the three regions are not concerted, and the very small chemical shift changes observed in L1, when mutants are made in L6 (Fig. 3*B* and *SI Appendix*, Fig. S5*A*), indicate that potential correlations are weak. With these simple assumptions, a calculation of the macroscopic catalytic rate is possible using only three additional rate constants, that is, *k*_on_, which is the microkinetic on-rate for the substrate, *k*_off_, which is the microkinetic off-rate; assumed to be the same for both product and substrate (*Methods*), and *k*_H_, which is the microkinetic rate for the hydrolysis (Fig. 4*B*).

In total, 93 states were considered (Fig. 4*B* and *SI Appendix*, Table S4), which correspond to 18 conformations for each of the five enzymatic species {E, ES, EP_lys_P_ace_, EP_lys_, EP_ace_}, a substrate species (S), an acetate product (P_ace_), and a lysine product (P_lys_). These 93 states are connected with the rate constants, *k*_on_, *k*_off_, and *k*_H_, (between enzymatic species) and with the rates derived from MQ-CPMG data (within the enzymatic species). We rationalized that hydrolysis only takes place when substrate-bound HDAC8, ES, is in the conformation with all the regions in their bound state, that is L1 in $A_{L1}^{TCSA}$   , L2,6 in $A_{L2,6}^{TCSA}$   and CAT in A, since this state is known to have an active site conformation competent for hydrolysis (39, 52). Moreover, based on the MQ-CPMG data (Fig. 2*G*), the dissociation of the acetate product takes place only when the L1 region is in the $C_{L1}^{}$ conformation.

For a given model that describes which conformations are involved in substrate association/dissociation and product dissociation, the concentration of each of the 93 states as a function of time can be calculated by integrating the underlying differential equations, see *Methods*. Specifically, with an initial scenario of only free enzyme, E, and substrate, S, present, one can calculate d[P_lys_]/d*t*, the enzymatic rate, as well as apparent Michaelis–Menten parameters, *k*_cat_ and K_M_ by repeating the calculation for different values of [S]. Using a similar model as described above, but only including E and E:TCSA (*k*_H_ = 0), it is also possible to calculate a macroscopic dissociation constant, K_d_, for an inhibitor and the macroscopic off-rate for that inhibitor, *k*_off,TCSA,macro_, which includes an additional microkinetic model parameter, *k*_off,TCSA_. In what follows, the experimentally determined Michaelis–Menten parameters (*k*_cat_ and K_M_ in H_2_O and *k*_cat_/K_M_ in D_2_O), the measured K_d_, and the observed macroscopic off-rate, *k*_off,TCSA,macro_ for TCSA for wild-type HDAC8 and for the L179A mutant were used in a least-squares analysis, where *k*_on_, *k*_off_, *k*_off,TCSA_, and *k*_H_ were determined, see Methods. A conformational selection (53) model was first tested, where association and dissociation take place in the conformation at which hydrolysis takes place, i.e., L1 in the bound state ( $A_{L1}^{TCSA}$   ), L2,6 in the bound state ( $A_{L2,6}^{TCSA}$   ), and CAT in the major state (A). The corresponding least-squares analysis resulted in a reduced χ^2^, χ^2^_red_ of 5.8 showing that such a model is unlikely.

Previous MD simulations(4) have demonstrated that the active site is substantially exposed in some of the sampled conformations. These conformations would therefore be excellent candidates for dissociation of the substrate and the lysine product. We therefore considered all possible models, where the substrate can bind and dissociate from two conformations, while the product can dissociate from one (group of) conformations(s). Making this consideration yielded two models with similar and appropriate reduced chi-squared values, χ^2^_red_ = 1.11 and χ^2^_red_ = 1.14 (*SI Appendix*, Table S5). The best-fit model corresponds to substrate association and dissociation from a conformation with the L1 in the bound state ( $A_{L1}^{TCSA}$   ), L2,6 in the $C_{L2,6}^{TCSA}$   state, and CAT in the B state. The conformation with L1 in the bound state, L2,6 in the free state ( $B_{L2,6}^{TCSA}$   ), and CAT in the B state is the only conformation from which the lysine product can dissociate from (Fig. 4 *B* and *C*). The only difference of the second-best model is that the lysine product can dissociate from $C_{L2,6}^{TCSA}$   . The analysis is summarized in *SI Appendix*, Table S5 and Fig. S7*A*, where the *P*-values between the top models are shown. Apart from the two models mentioned above, only a small fraction of other models (70 of the 2,304 tested) give a *P*-value larger than 0.05.

To cross-validate the model determined above, a second mutant M274A-HDAC8 was produced, and MQ-CPMG relaxation dispersions, binding experiments to TCSA, and enzymatic assays were performed. Briefly, the MQ-CPMG experiments show that the M274A mutant samples conformations that are similar to those sampled by wild-type HDAC8 as shown by the linear correlations between the |Δω| shifts (*SI Appendix*, Fig. S*8* *A*–*C*). Least-squares analysis of the MQ-CPMG data for M274A-HDAC8 showed a drastic decrease in the population of the $B_{L2,6}^{free}$   state, which resembles the bound conformation, by a factor of ~40 (*p*_B_ changes from 10 to 0.25%) compared to wild-type (*SI Appendix*, Fig. S8*E*). Moreover, the M274A mutant had substantial reduced enzymatic activity of 3 ± 2% (>30 times lower *k*_cat_/K_m_) compared to wild-type HDAC8 and also shows a substantial decrease in binding affinity to TCSA from K_d_ of 5 ± 2 µM to 116 ± 12 µM (*SI Appendix*, Fig. S8*G*). When the best-fit model, derived from only wild-type HDAC8 and L179A-HDAC8 data, is used with the intrinsic dynamics derived from the M274A mutants, inhibitor potency and enzyme activity are in excellent agreement with those obtained experimentally. Specifically, a K_d_ of 110 μM is predicted vs. 116 ± 12 μM measured, an inhibitor off-rate of 0.86 s^−1^ is predicted vs. 1.19 ± 0.15 s^−1^ measured. It should be stressed that while both the wild-type HDAC8 and L179A-HDAC8 have substantial slower macroscopic inhibitor off-rates, *k*_off,TCSA,macro_ of 0.13 and 0.19 s^−1^, respectively, the intrinsic dynamics of M274A-HDAC8 and the model still predict a correct inhibitor off-rate for the M274A mutants. Finally, the model and intrinsic dynamics of M274A-HDAC8 also predicts a very low enzymatic activity with *k*_cat_/K_M_ of 0.57 M^−1^s^−1^ vs. 1.2 ± 1.0 M^−1^s^−1^ measured experimentally. Finally, a cross-validation χ^2^ was used to test whether any of the other models in *SI Appendix*, Table S5 can accurately explain the data of the M274A-HDAC8 mutant. The cross-validation χ^2^ was calculated from the calculated and experimentally observed *k*_cat_/K_M_, K_d_, and *k*_off,In,macro_ for the M274A-HDAC8, using ∑_i_ (*obs*_i_ − *calc*_i_)/σ_i_, where σ is the experimental uncertainty. The model identified above, model 1, has a cross-validation χ^2^ of 5.5 (reduced cross-validation χ^2^ of 1.8), and the second-best model has a cross-validation χ^2^ of 5.4, which are highly probable. All other models tested had cross-validation χ^2^ above 13.7. The two models identified (Fig. 4*C*), therefore, describe the data significantly better than any of the other 2,302 models (*SI Appendix*, Fig. S7*B*).

The optimized parameters themselves, obtained in the least squares fit, also provide insight into the function of HDAC8. For example, the two models identified, because of their similar nature, both indicate nearly diffusional limited binding with *k*_on_ = 4.0 ± 1.3 μM^−1^ s^−1^ as well as a very weak interaction, comparable to a couple of hydrogen bonds, between the substrate and the enzyme in the binding competent state, with *k*_off_ of 5.1 × 10^5^ ± 1.7 × 10^5^ s^−1^ and 6.9 × 10^5^ ± 2.3 × 10^5^ s^−1^, respectively. Similarly, an identical microkinetic off-rate of *k*_off,I_ = 124 ± 40 s^−1^ for the inhibitor is obtained for both models. Finally, the microkinetic hydrolysis rate, *k*_H_(H_2_O), is 2.04 ± 0.16 s^−1^ for the best-fit model (model 1) and 2.9 ± 0.3 s^−1^ for the second-best model (model 2), which are close to the experimentally measured *k*_cat_ (0.90 ± 0.09 s^−1^) for wild-type HDAC8, and the reaction rate is therefore to some extent limited by hydrolysis at least in wild-type HDAC8.

Overall, in each of the two best-fit models shown in Fig. 4*B*, only one set of microkinetic rate constants, *k*_on_, *k*_off_, *k*_H_, (*k*_H_(H_2_O) = 8.8 × *k*_H_(D_2_O)), and *k*_off,TCSA_, accurately describes the Michaelis–Menten parameters, the TCSA affinity, K_d_ and inhibitor off-rate, *k*_off,TCSA,macro_, for both wild-type HDAC8, and for the mutants L179A and M274A. In turn, this means that for HDAC8, the intrinsic dynamics alone dictate the change in both enzymatic activity and inhibitor binding/dissociation upon introduction of single-point mutations.

## Discussion

In the era of computational structural predictions (54, 55), the prevalence of protein flexibility and heterogeneity has become increasingly clear. However, the detailed functional role of sparsely populated and transient conformations of enzymes often remains elusive, although it is well known that these conformations exist and that they are imperative for function (1, 2, 26, 56). Here, we used a comprehensive set of methyl-TROSY MQ-CPMG data together with inhibitor-binding and enzymatic assays to pinpoint the functional roles of sparsely populated states of the enzyme HDAC8. Our results show that substrate association and dissociation as well as product dissociation are only possible from specific conformations of HDAC8, conformations that are sparsely populated in free HDAC8 and not visible in current structures. Moreover, our results also show that the intrinsic structural dynamics (kinetics and thermodynamics) between the intrinsically sampled conformations dictate the enzymatic activity and binding affinity of HDAC8 to the inhibitor TCSA. That is, the change in intrinsic dynamics observed upon introducing mutations in HDAC8 accurately accounts for the altered enzymatic activity of these mutants as well as their decreased affinity and decreased residence-time for the TCSA inhibitor.

The enzymatic activity and inhibitor binding of mutant enzymes, in particular for mutations outside a catalytic site, are often interpreted in the framework of altered network of interactions between the enzyme and the substrate or inhibitor. Such rational interpretations are often based on crystal structures of the enzyme and therefore based on interactions only present within one conformation. In contrast, our results show that at least in some cases, it is the altered intrinsic dynamics caused by the mutants that can account accurately for the changes to function. Similarly, our results set out a framework for understanding the effect of missense mutants, for example, CdLS mutants of HDAC8, since many of these are expected to alter the intrinsic dynamics between sampled conformations. For HDAC8, mutants near the L2 and L6 loops are expected to affect substrate binding and lysine product dissociation, as seen for the L179A and M274A mutants that lead to substantially lower affinities for the inhibitor and larger apparent Michaelis–Menten constants relative to the wild-type HDAC8. Mutants near the L1 loop of HDAC8 mainly affect the dissociation of the acetate product and thus the apparent turnover rate *k*_cat_.

Pinpointing the functional roles of transient conformations of enzymes provides detailed mechanistic insight into enzymatic function and can inform our understanding of the effect of missense mutations related to disease. Our study demonstrates that changes to intrinsic unimolecular dynamics upon mutation can fully account for altered enzymatic activity, which has significant implications for interpreting disease-causing mutants. Additionally, the ability to accurately predict altered inhibitor potencies, solely from intrinsic dynamics, has the potential to improve drug developments since the conformations responsible for inhibitor association and dissociation can differ from the ground state, which is most often observed in the available crystal or computationally predicted structures. Whereas stabilizing the ground state of an inhibitor-bound conformation can increase the affinity, the potency of many drugs is predicted by the residence time (57), or off-rate, which, as we have now shown, can be dictated by the effective rate from the ground state to the state implicated with dissociation.

## Materials and Methods

### Protein Expression, Purification, and Mutagenesis.

The human HDAC8 construct described by Vannini et al. (39) with a C-terminal hexa-histidine tag in pET21b expression vector was used to express the protein in BL21(λDE3) cells. The cells were grown at 37 °C in ~99% D_2_O minimal M9 media containing 1 g/L ^15^NH_4_Cl as the sole nitrogen source and 3 g/L [^2^H/^12^C]-glucose as the sole carbon source. Methyl labeling of Ile, Leu, Val, and Met in one sample was achieved by the addition of 60 mg/L alpha-ketobutyric acid [U-^12^C/^2^H, methyl-^13^CH_3_] (for labeling of isoleucines), 90 mg/L α-ketoisovaleric acid [U-^12^C/^2^H, methyl-(^13^CH_3_,^12^CD_3_)] (for labeling of valine and leucine residues), and 150 mg/L of methionine [^13^C/^1^H] (for labeling of methionine residues). To achieve the U-[^12^C, ^2^H]-LV-[^13^CH_3_]_2_ double methyl labeling of valine and leucine for the 3D-HMBC-HMQC experiment, 80 mg/L of {U-[^12^C,^2^H] [^13^CH_3_]_2_} α-ketoisovaleric acid was used. To prepare the valine-only sample, 80 mg/L of α-ketoisovaleric acid [U-^12^C^2^H, methyl-(^13^CH_3_)_2_] and α-ketoisocaproic acid [U-^12^C^2^H] isopropyl-^2^H_7_ were used. HDAC8 sample with U-[^2^H], U-[^12^C], Leu/Val-[^13^C^1^H_3_]pro-S stereospecific labeling was prepared by adding 2-[^13^C]methyl-4-[^2^H_3_] acetolactate. All precursors for the different labeling were added 1 h prior to induction. Expression was induced at an OD_600nm_ between 0.7 to 1.0 by addition of 0.5 mM ITPG and 0.2 mM ZnCl_2_ at 21 °C for 16 to 18 h. In this study, the following four different methyl-labeled samples were used: 1) ILVM (Ile,Leu, Val, Met)-labeled sample with {Ile (^13^C^δ1^H_3_), Leu(^13^CH_3_,^13^CD_3_), Val(^13^CH_3_,^13^CD_3_)}, Met(^13^CH_3_) [U-^15^N,^12^C,^2^H] labeling; 2) double methyl-labeled ILV sample- with {Ile(^13^C^δ1^H_3_), Leu(^13^CH_3_,^13^CH_3_), Val(^13^CH_3_,^13^CH_3_)} [U-^15^N,^12^C,^2^H] labeling; 3) valine-only sample with Val(^13^CH_3_,^13^CH_3_)} [U-^15^N,^12^C,^2^H] labeling; and 4) proS sample with {Ile(^13^C^δ1^H_3_), Leu(^13^CH_3_^δ2^,^13^CD_3_^δ1^), Val(^13^CH_3_^γ2^,^13^CD_3_^γ1^) } [U-^15^N,^12^C,^2^H] labeling.

The cell pellet was collected by centrifugation and resuspended in lysis buffer containing 50 mM Tris–HCl pH 8.0, 3 mM MgCl_2_, 500 mM KCl, 10 mM imidazole, 5% glycerol, and 10 mM β-mercaptoethanol. The cells were lysed by sonication after addition of 0.25% IGEPAL, small amounts of DNAse, lysozyme, and protease inhibitors tablets (1 tablet per 50 mL, Roche). The lysate was centrifuged at 18 k rpm for 1 h. After cell lysis protein was purified by Ni-NTA affinity chromatography using a linear imidazole gradient (10 to 250 mM imidazole in lysis buffer). Fractions containing HDAC8 were pooled and concentrated by ultrafiltration through 10 kDa cutoff Amicon (Millipore) ultrafiltration membranes. Further, a size-exclusion chromatography on Superdex-75 column was carried out in buffer containing 50 mM Tris pH 8.0, 150 mM KCl, 1 mM TCEP, and 5% glycerol. Fractions containing purified HDAC8 were pooled together and concentrated by 10 kDa cutoff Amicon (Millipore) ultrafiltration membranes. The concentrated sample was buffer exchanged into NMR buffer (50 mM K_2_HPO_4_ pH 8.0, 30 mM KCl, 4 mM DTT, and 1 mM NaN_3_). The yield of purified protein was 1.5 to 2 mg/L of culture medium. Mutations were introduced by the QuikChange approach, and their sequences were verified by DNA sequencing**.**

### NMR Data Acquisition.

Most of the NMR experiments of wild-type HDAC8 and mutant HDAC8 were acquired in NMR buffer containing 50 mM K_2_HPO_4_ pH 8.0, 30 mM KCl, 4 mM DTT, and 1 mM NaN_3_ in 100% D_2_O. NMR experiments mentioned in the manuscript were recorded on Bruker 600, 700, 800, and 950 MHz spectrometers equipped with cryogenic TCI probes. All NMR spectra were processed using NMRPipe and analyzed with CARA and CCPN (58–60). Two-dimensional methyl-TROSY spectra were recorded using the experiment described by Tugarinov et al. (13). The 3D-HMBC-HMQC experiment (61) was acquired with 1,024, 48, and 64 complex points in the ^1^H, ^13^C_HMQC_, and ^13^C_HMBC_ dimensions with spectral widths of 13, 16, and 13 ppm, respectively. The 3D ^13^C HMQC-NOESY-^13^C HMQC experiment [^13^C_methyl_(ω_1_)–NOESY–^13^C_methyl_(ω_2_)–^1^H_methyl_(ω_3_)] for chemical shift assignments was performed with 1024, 56, and 88 complex points in the ^1^H, ^13^C_hmqc_, and ^13^C_noesy_ dimensions with spectral widths of 11.7, 26.15, and 26.15 ppm, respectively, using standard pulse sequences at static magnetic fields of 18.8 T with a NOESY mixing time of 400 ms. The 4D HMQC-NOESY-HMQC spectra were recorded using a 2% non-uniform sampling schedule generated with a Poisson Gap distribution (62). Following this, the spectra were reconstructed using the iterative soft thresholding protocol (63). The reconstruction was carried out on the Legion cluster at UCL, and processing with nmrPipe was carried out on the NMRbox server (64). Methyl-TROSY-based MQ-CPMG experiments were performed at different field strengths and at three different temperatures (298 K, 288 K, and 278 K) according to Korzhnev et al. (17) using a constant-time delay for the CPMG period of 30 ms and CPMG frequencies from 20 to 1,000 Hz (17). CPMG relaxation dispersion experiments on an HDAC8 sample with excess amounts of TCSA were performed at 1:3 molar ratio. Similarly, an additional CPMG relaxation dispersion experiment was acquired in NMR buffer with 95% H_2_O+5% to assess the kinetic solvent isotope effect on internal dynamics of wild-type HDAC8.

### Chemical Shift Assignment of the ILVM Methyl Groups of HDAC8.

The structure-based assignment method was used for the assignment of the methyl resonances in the methyl-TROSY NMR spectrum of the ILVM-labeled sample. The first step of the structure-based methyl group assignment was the identification of methyl groups of different amino acid types. In the ILVM spectrum, the methyl resonances of Ile and Met residues were readily identified by examining proton and carbon chemical shifts as they occupied in different isolated regions of the spectrum. Despite the fact that valine residues have a smaller ^13^C shift compared to leucine residues, there is no clear spectral division between them. As a result, the methyl resonances of Leu and Val residues cannot be directly identified. Methyl resonances of valine and leucine residue were identified by comparing the LV region of ILVM spectrum to the methyl spectrum of a valine-only sample (see above). Afterward, two intraresidual methyl groups of Leu and Val were linked by a 3D-HMBC-HMQC experiment (61), recently developed by our group, and 4D HMQC-NOESY-HMQC, acquired on double methyl-labeled sample. Later, 4D HMQC-NOESY-HMQC and 3D ^13^C HMQC-NOESY-^13^C HMQC experiments were used to assign the methyl resonances. The assignment of methyl group of 23 Ile and two methionine has already been reported in our previous work (3). Methyl resonances of 7 Met, 23 Val, and 31 Leu were easily assigned by comparing the methyl-methyl NOE network with the methyl–methyl distance network of the crystal structure of HDAC8 (PDB: 1T64). Methyl resonances of V61, L179, M274, V214, and V217 were assigned by site directed mutagenesis to complete the assignment. The stereospecific assignment of Leu and Val was achieved by comparing the methyl TROSY spectrum of *proS* sample with the LV spectrum. This combined approach of site-directed mutagenesis and structure-based methyl-methyl NOE assignment allowed the unambiguous assignment of 23 methyl resonances of 25 Ile, 52 resonances of 26 Val, 64 resonances of 32 Leu, and 10 methyl resonances of 10 Met.

### Analysis of MQ-CPMG Relaxation Dispersion Data.

Peak intensities of the methyl-TROSY-based MQ-CPMG relaxation dispersion data were obtained using FuDA of the methyl-TROSY-based MQ-CPMG relaxation dispersion data (65). The effective transverse relaxation rates (*R*_2,eff_) for each methyl group at different temperatures were calculated as *R*_2,eff_(ν) = −ln(*I*_ν_/*I*_0_)/*T*_relax_, where *I*_ν_ is the peak intensity at the CPMG frequency ν and *I*_0_ the intensity measured without the delay *T*_relax_. These rates were used as input for least-squares fitting using ChemEx (https://github.com/gbouvignies/chemex) to extract the thermodynamic parameters and the ^13^C chemical shift differences. In the least-squares analysis, the proton Δω were all fixed to the very small value of 0.01 ppm. Nine small, yet significant, ^1^H relaxation dispersions were observed in ^1^H methyl relaxation dispersions (66) of HDAC8:TCSA, however, including these in the least-squares analysis did not affect the derived ^13^C Δω (*R* = 0.99). The thermodynamic parameters include (for a two-site exchange) ΔH_B_, ΔH^‡^, ΔS_B_, and ΔS^‡^, where ΔH_B_ and ΔS_B_ are the enthalpy and entropy of the sparse-state B relative to A, and ΔH^‡^ and ΔS^‡^ are the enthalpy and entropy of the transition state relative to A. The ^13^C chemical shift difference (Δω) is between the major and minor conformations, a residue/resonance-specific parameter (3, 17, 67). Errors in the extracted exchange parameters were estimated by using Monte Carlo simulations. Data for free HDAC8, L179A-HDAC8, M274A-HDAC8, and HDAC8:TCSA were analyzed using either a two-site exchange model or a three-site exchange model using the Eyring equation for temperature-dependent studies (68). For analyzing data over multiple temperatures, Δω values were assumed to be temperature independent. Within the three-site analysis, two models were considered: the bifurcated model (B ⇌ A ⇌ C) and the linear model A ⇌ B/C ⇌ C/B. In free HDAC8, L179A-HDAC8, and M274A-HDAC8, minimum χ^2^_red_ values were obtained for the bifurcated model, while for HDAC8:TCSA, minimum χ ^2^_red_ values were obtained for the linear model (*SI Appendix*, Table S2).

### NMR Titration with Inhibitor.

To compare the binding affinity of wild-type HDAC8 and mutant-HDAC8s for substrate/inhibitor, NMR titrations of wild-type/mutant HDAC8 with TCSA were performed using 2D methyl-TROSY (^13^C^1^H HMQC) experiment. The shift changes (ΔCS) upon TCSA binding were calculated as $\begin{array}{ccc} ∆\mathrm{CS} & = & \sqrt{{\left({\Delta \delta }_{H}/\alpha \right)}^{2}+{\left({\Delta \delta }_{C}/\beta \right)}^{2}} \end{array}$ , (69) where α and β are standard deviation of the ^1^H and ^13^C chemical shifts for methyl groups, deposited in the Biological Magnetic Resonance Data Bank. Separate values of α and β were used for different methyl groups.

Upon addition of TCSA at substoichiometric concentration, a few cross-peaks in the methyl-TROSY spectrum split into two fractions, and further addition of TCSA resulted in an increase in the intensity of new peaks (bound state), while decreasing the intensity of peaks representing the unbound state. It suggests that inhibitor binding is an intermediate-to-slow exchange process. Therefore, in order to obtain K_d_ values, the TITAN 2D lineshape analysis program(70) was used within NMRbox (64), which accounted for both CSPs and line broadening (71). For all wild-type and mutant HDAC8s, single-site binding model (two-state ligand binding in TITAN) was used. The K_d_ values were estimated using global fitting of peaks from a total of 10 to 15 methyl resonances. Later, errors were evaluated using bootstrap analysis. Results from the analysis of TCSA binding are shown in *SI Appendix*, Table S5. Additionally, we have compared the binding affinity of TCSA with wild-type HDAC8 in 100% D_2_O and 95% H_2_O + 5% D_2_O to observe the kinetic isotope effect on binding affinity, wherein no significant change in K_d_ was observed.

### Activity Assay of HDAC8 and Its Mutants.

The MAL (Boc-Lys(Ac)-7-amino-4-methylcoumarin) assay was performed as described previously(43) with slight modifications to characterize the activity of wild-type HDAC8, L179A-HDAC8, and M274A-HDAC8 using MAL as the substrate. In this assay, the aliquots of wild-type HDAC8 and each mutant were prepared at 0.2, 0.4, 0.6, and 0.8 μM concentrations in the total reaction volume of 50 μL in assay buffer (50 mM Tris pH 8.0, 137 mM NaCl, 2.7 mM KCl, 1 mM MgCl_2_, and 1 mg/ml Bovine Serum Albumin). The stock solution of the MAL substrate (50 mM in dimethyl sulfoxide) was diluted in assay buffer having enzyme solution to yield a final concentration of 200 μM in the reaction volume. The HDAC8:MAL solutions having different concentrations of the enzyme and similar concentrations of MAL were incubated at 25 °C. Afterward, 50 μL of the reaction solution was pipetted on a 96-well white NBS microplate preloaded with 50 μL developer solution (10 mg/mL trypsin and 4 μM DCPI in assay buffer) at 0, 10, 20, 30, 40, 50, and 60 min. The fluorescence was measured (excitation = 380 nm and emission = 460 nm) after 30 min incubation at 25 °C on a BMG FLUOstar Optima plate reader. Errors (rmsd) in relative enzymatic activities were estimated as the rmsd of the activity measured in assays for wild-type HDAC8 and each mutant (six time points and four substrate concentrations). Likewise, the MAL activity assay was performed at five different concentrations of D_2_O (0%, 12.5%, 25%, 50.0%, and 100%) to observe the kinetic isotope effect on the biochemical activity of HDAC8.

#### Analysis of Enzymatic Activity and Inhibitor Binding Using Rates Derived from CPMG Relaxation Dispersion.

In order to simulate numerically the macroscopic enzymatic activity and macroscopic inhibitor binding of HDAC8 and its mutants from the rates derived from CPMG relaxation, 93 states were defined (*SI Appendix*, Table S4). State 1-18 correspond to the 18 states (Fig. 4*C*) of free HDAC8 (E) defined by the CPMG relaxation dispersions, state 19-36 correspond to substrate-bound (or inhibitor-bound) HDAC8 (ES), state 37-54 correspond to HDAC8 bound to both products, acetate and lysine (EP_lys_P_ace_), state 55-72 correspond to HDAC8 bound to acetate product (EP_ace_), and state 73-90 correspond to HDAC8 only bound to lysine product (EP_lys_). State 91 was the substrate (S), state 92 the lysine product (P_lys_), and state 93 the acetate product (P_ace_). The differential equations describing the dynamics of the system were setup and solved numerically using the scipy (72) function odeint.

To ensure detailed balancing (73) of the rate equations, when binding of the substrate could take place at multiple states within free HDAC8, e.g., E*_i_* and E_j_, the binding and dissociation reactions were defined by:

$$\begin{array}{c} \frac{d\left[{ES}_{i}\right]}{\mathrm{dt}}=k_{on}\left[S\right]\left[E_{i}\right]\frac{p({ES}_{i})}{p\;\left({ES}_{i}\;\right)+p\left({ES}_{j}\right)}\\ \;-k_{off}\left[ES_{i}\right]\frac{p(E_{i})}{p\;\left(E_{i}\;\right)+p\left(E_{j}\right)}, \end{array}$$

$$\begin{array}{c} \frac{d\left[{ES}_{j}\right]}{\mathrm{dt}}=k_{on}\left[S\right]\left[E_{j}\right]\frac{p({ES}_{j})}{p\;\left({ES}_{i}\;\right)+p\left({ES}_{j}\right)}\\ \;-k_{off}\left[ES_{j}\right]\frac{p(E_{j})}{p\;\left(E_{i}\;\right)+p\left(E_{j}\right)}, \end{array}$$

where *k*_on_ and *k*_off_ are the microkinetic parameters determined by least-squares fitting. For example, when substrate can bind to the two states *i* = {L1=Bound, L2,6=C_L2,6_, CAT=B} and *j* = {L1=Bound, L2,6=Free, CAT=B}, the above equations ensure that there is a detailed balancing of the rate equations and therefore no flux of the system at equilibrium.

Hydrolysis was included by a first-order reaction rate constant, *k*_H_, from ES (L1 = Bound, L2,6 = Bound, CAT = A) to EP_lys_P_ace_ (L1 = Bound, L2,6 = Bound, CAT=A). Dissociation of the acetate product was included by a first-order rate constant, *k*_off,ace_ from EP_lys_P_ace_ (L1=C_L1_) to EP_lys_ (L1=C_L1_) and from EP_ace_ (L1=C_L1_) to E (L1= C_L1_). It was assumed that *k*_off,ace_ had reached the diffusion limit, *k*_off,ace_ = 10^6^ s^−1^. Similarly, dissociation of the lysine product was included by a first-order off-rate, *k*_off,P_ from EP_lys_P_ace_ and EP_lys_. To the differential equations were added the unimolecular rates obtained from the CPMG relaxation dispersion experiments. Specifically, the unimolecular rates within E and EP_ace_ were taken from the analysis of the CPMG relaxation dispersions of free HDAC8, whereas the unimolecular rates within ES, EP_lys_P_ace_, and EP_lys_ were taken from the analysis of the CPMG relaxation dispersion of TCSA-bound HDAC8. The MQ-CPMG data recorded in D_2_O were used for both wild-type HDAC8 in D_2_O and in H_2_O because the data obtained in H_2_O were associated with substantial larger uncertainties and comparisons showed that there are no significant differences (*SI Appendix*, Fig. S6 *C*–*F*). A succinct description of the python function used to define the differential equations is provided in supporting material.

To model enzyme activity, the initial state had concentrations of state 19-90, 92, and 93 to zero, the concentration of state 91 equal to the substrate, [S]_0_, and states 1-18 (E) were given concentrations equal to the populations derived from the CPMG data multiplied by the macroscopic enzyme concentration E_0_ = [HDAC8] (*t* = 0). The numerical integration was initiated over a grid of 60,000 logarithmically spaced time points between 1 μs and 600 s. It should be noted that the odeint function automatically switches between stiff and nonstiff integration methods and therefore provides a robust integration. The macroscopic initial steady-state rate, *v*_0_, was obtained by calculating the gradient of the concentration of state 92 (P_lys_) with respect to time, *v*_0_ = d[P_lys_]/d*t*, from 60 s to 600 s as was done in the experiments. The simulation was run for 50 substrate concentrations, [S]_0_, between 0 mM and 250 mM. Finally, the apparent *k*_cat_ and K_M_ parameters were obtained by a least-squares fit of *v*_0_ vs. [S]_0_ to the classical Michaelis–Menten equation, *v*_0_ = [E]_0_*k*_cat_[S] / ([S] + K_M_).

Inhibitor binding and dissociation were modeled in a similar manner by setting *k*_H_ = 0, and the macroscopic K_d_ was calculated after equilibrium had been established as [E][TCSA]/[E:TCSA]. Subsequently, using the equilibrium inhibitor-bound state, the inhibitor macroscopic off-rate, *k*_off,TCSA,macro_, was calculated by setting [S] = 0 mM for the equilibrated system and fitting [E:TCSA](*t*) to an exponential decay of the form [E:TCSA](*t*) = *A* exp(−*k*_off,TCSA,macro_ *t*). For each of the possible kinetic models, a least-squares fit was performed to determine *k*_on_, *k*_off,TCSA_, *k*_off,S_ = *k*_off,P_ and *k*_H_. The off-rate for acetate was fixed at 10^6^ s^−1^. The experimental constraints were K_M_ and *k*_cat_ for wild-type HDAC8 in H_2_O, *k*_cat_/K_M_ for wild-type HDAC8 in D_2_O, *k*_cat_/K_M_ for L179A-HDAC8, and K_d_ and *k*_off,TCSA,macro_ for the inhibitor for wild-type HDAC8 and the L179A mutant (*SI Appendix*, Table S5). A five-parameter fit was also performed, with different off-rates for the product (*k*_off,P_) and the substrate (*k*_off,S_), thus optimizing {*k*_on_, *k*_off,TCSA_, *k*_off,S_, *k*_off,P_, and *k*_H_}. The five-parameter fit led to the essentially same χ^2^ (4.4261 vs. 4.4264) as the four-parameter fit, but larger uncertainties of the derived parameters.

## Supplementary Material

Appendix 01 (PDF)

Dataset S01 (XLSX)

Dataset S02 (XLSX)

Dataset S03 (XLSX)

Dataset S04 (XLSX)

Dataset S05 (XLSX)

Dataset S06 (XLSX)

Dataset S07 (XLSX)

Dataset S08 (XLSX)

## Data Availability

All study data are included in the article, and supporting information. Raw NMR data used for the analysis are available on Zenodo. https://doi.org/10.5281/zenodo.8348964 (74).
