# Supplementary material for: Intrinsic structural dynamics dictate enzymatic activity and inhibition
Source: Proc Natl Acad Sci U S A. 2023 Oct 2;120(41):e2310910120. doi: 10.1073/pnas.2310910120 (PMC10576142; doi:10.1073/pnas.2310910120)
Supplement: Supplementary file 1 — Appendix 01 (PDF) [file pnas.2310910120.sapp.pdf]

PNAS

Supporting Information for

Intrinsic structural dynamics dictate enzymatic activity and inhibition

Vaibhav Kumar Shukla, Lucas Siemons, D. Flemming Hansen*

*) D. Flemming Hansen, Email: d.hansen@ucl.ac.uk

Datasets included:

Dataset S1:	Raw data for Figure 1
Dataset S2:	Raw data for Figure 2
Dataset S3:	Raw data for Figure 3
Dataset S4:	Raw data for Figure S2
Dataset S5:	Raw data for Figure S4
Dataset S6:	Raw data for Figure S5
Dataset S7:	Raw data for Figure S6
Dataset S8:	Raw data for Figure S8

This PDF file includes:

Supplementary Methods
Figures S1 to S8
Tables S1 to S5
Supplementary References (1–3)

Supporting Information Text

Supplementary Methods

Solvent deuterium kinetic isotope effect. The microkinetic hydrolysis of HDAC8 has previously been suggested to be rate-limited by the nucleophilic attach of H_2O (see main text), which in turn comprises the break of a H-O bond in a water molecule. Assuming an average H-O stretch vibration in H_2O with an energy of 3350 cm^{-1} (1) corresponds to a frequency of $\nu_{HO} = 9.9\times10^{13}$ Hz. The corresponding stretch vibration in D_2O will therefore have a frequency of

$$\nu_{\mathrm{DO}} = \sqrt{\left(\frac{m_{\mathrm{H}} m_{\mathrm{O}}}{m_{\mathrm{H}}+m_{\mathrm{O}}}\right) / \left(\frac{m_{\mathrm{D}} m_{\mathrm{O}}}{m_{\mathrm{D}}+m_{\mathrm{O}}}\right)}\ \nu_{\mathrm{HO}} = \sqrt{\frac{16}{17} / \frac{32}{18}}\,\nu_{\mathrm{HO}} = 7.2 \times 10^{13}\ \mathrm{Hz}$$

where m_H (1.008 u) is the mass of the proton, m_D (2.014 u) is the mass of deuterium, and m_O (15.999 u) is the mass of oxygen. Subsequently, we can estimate the solvent deuterium kinetic isotope effect on the micro-kinetic hydrolysis rate, k_H, using classical rate theory:

$$\frac{k_{\mathrm{H}}(\mathrm{H_2O})}{k_{\mathrm{H}}(\mathrm{D_2O})} = \exp\left(\frac{h(\nu_{\mathrm{HO}} - \nu_{\mathrm{DO}})}{2\,k_{\mathrm{B}}T}\right) = 8.79\ \approx 8.8$$

where h is Planck's constant, k_B is Boltzmann's constant, and T is the absolute temperature.

Analysis of enzymatic activity and inhibitor binding

Below is a succinct version of the python code used to calculate the numerical derivatives of the concentration of the 93 states. The definitions of the 93 states are provided in Supplementary Table S3 and these derivatives are integrated using the scipy *odeint* function to obtain the concentration of all 93 states as a function of time.

```
def calc_dydt_gen(conc,time, FreeK, BoundK):
    """
    conc   is an array of the current 93 concentrations
    FreeK  is an 18x18 matrix holding the internal rate constants obtained
           from analysis of the CPMG data of free HDAC8
    BoundK is an 18x18 matrix holding the internal rate constants obtained
           from analysis of the CPMG data of Inhibitor-bound HDAC8
    FreeP  is a 18x1 vector holding the equilibrium populations of
           free HDAC8
    BoundK is a 18x1 vector holding the equilibrium populations of
           bound HDAC8

    D. Flemming Hansen, June 2022
    """
    binding_sub = (12,15)
    release_lys = (12)
    release_ace = (2,5,8,11,14,17)
    #
    # Initialise the vector dydt, which holds all the derivatives
    dydt = zeros(5*18+3)   #{ E, ES, EPacePlys, EPace, EPlys }, S, Plys,
Pace
    #
    # Add internal dynamics for E; state {0,...,17}
    for s in range(18):
        for source in range(18):
```

```
        dydt[s]      += FreeK[s,source] * conc[source]
        dydt[source] -= FreeK[s,source] * conc[source]
#
# Add internal dynamics for ES; state {18,...,35}
for s in range(18):
    for source in range(18):
        dydt[s+18]      += BoundK[s,source] * conc[source+18]
        dydt[source+18] -= BoundK[s,source] * conc[source+18]
#
# Add internal dynamics for EPacePlys; state {36, ..., 53}
for s in range(18):
    for source in range(18):
        dydt[s+2*18]      += BoundK[s,source] * conc[source+2*18]
        dydt[source+2*18] -= BoundK[s,source] * conc[source+2*18]
#
# Add internal dynamics for EPace; state {54, ... 71}
for s in range(18):
    for source in range(18):
        dydt[s+3*18]      += FreeK[s,source] * conc[source+3*18]
        dydt[source+3*18] -= FreeK[s,source] * conc[source+3*18]
#
# Add internal dynamics for EPlys; state {72, ..., 89}
for s in range(18):
    for source in range(18):
        dydt[s+4*18]      += BoundK[s,source] * conc[source+4*18]
        dydt[source+4*18] -= BoundK[s,source] * conc[source+4*18]
#
# Hydrolysis
dydt[0+1*18]  -=  kH * conc[0+1*18]
dydt[0+2*18]  +=  kH * conc[0+1*18]
#
# Substrate binding
for state in binding_sub:
    # For detailed balancing we need the populations of the states
    # we are going to 'state + 18', which is 'state' in 'bound'
    weight = BoundP[state]/sum(BoundP[binding_sub])
    dydt[state+18] += weight * kon * conc[90] * conc[state]
    dydt[state]    -= weight * kon * conc[90] * conc[state]
    dydt[90]       -= weight * kon * conc[90] * conc[state]
#
for state in release_sub:
    # For detailed balancing we need the populations of the states
    # we are going to 'state', which is 'state' in 'free'
    weight = FreeP[state]/sum(FreeP[release_sub])
    dydt[state+18] -= weight * koffS * conc[state+18]
    dydt[state]    += weight * koffS * conc[state+18]
    dydt[90]       += weight * koffS * conc[state+18]
#
# Product Lys production
for state in release_lys:
    #
    # Product Lys production from EPlysPAce -> EPAce
    weight = FreeP[state]/sum(FreeP[release_lys])
    dydt[91]         += weight * koffPlys * conc[state+2*18]
    dydt[state+2*18] -= weight * koffPlys * conc[state+2*18]
    dydt[state+3*18] += weight * koffPlys * conc[state+2*18]
    #
```

```
        # Product Lys production from EPlys -> E
        dydt[91]         += weight * koffPlys * conc[state+4*18]
        dydt[state+4*18] -= weight * koffPlys * conc[state+4*18]
        dydt[state+0*18] += weight * koffPlys * conc[state+4*18]

    #
    # Product acetate dissociation
    for state in release_ace:
        # Product Ace production from EPlysPace
        weight = BoundP[state]/sum(BoundP[release_ace])
        dydt[92]         += weight * koffPace * conc[state+2*18]
        dydt[state+2*18] -= weight * koffPace * conc[state+2*18]
        dydt[state+4*18] += weight * koffPace * conc[state+2*18]
        #
        # Product Ace production from EPace
        weight = FreeP[state]/sum(FreeP[release_ace])
        dydt[92]         += weight * koffPace * conc[state+3*18]
        dydt[state+3*18] -= weight * koffPace * conc[state+3*18]
        dydt[state+0*18] += weight * koffPace * conc[state+3*18]

    return dydt
```

Approximate equations for inhibitor binding

The above function and numerical integration allow a simulation of the enzymatic activity of HDAC8 and the mutants L179A and M274A. Additional insight can be gained by deriving approximate equations that described the experimentally observed parameters as a function of the parameters obtained in the CPMG analysis and the four rates derived by least-squares fitting, k_{on}, $k_{off,I}$, $k_{off,S}$ = $k_{off,P}$ and k_H. As an example, an expression for the macroscopic K_d can be derived, where only states 0-35 are considered along with the inhibitor I. The effective macroscopic K_d is given by the concentrations at equilibrium:

$$K_d = \frac{[I][E]}{[EI]}$$

where [E] is the total concentration of free enzyme, which equals the sum of states 0-17 (Supplementary Table 3), [EI] is the concentration of the inhibitor-bound enzyme, which equals the sum of states 18-35. For the best-fit model, binding involves state 12 and state 15 of E (Supplementary Table 3), which forms state 30 and state 33 in EI. The on-rate to state 12, after applying detailed balancing, see *e.g.* (2), is: $k_{on,12}$ = k_{on} $p_{30}/(p_{30}+p_{33})$. The population p_{30} is the population of the first binding competent state within the EI complex (L1=Bound, L2,6=Free, CAT=B) and p_{33} is the population of the second binding competent state (L1=Bound, L2,6=C_{L6}, CAT=B). Similarly, $k_{off,I,12}$ = $k_{off,I}$ $p_{12}/(p_{12}+p_{15})$ and therefore,

$$\frac{[\text{state 12}]}{[\text{state 30}]} = \frac{k_{\text{off,I,12}}}{k_{\text{on,12}}[\text{I}]}$$

$$K_d = \frac{[I][E]}{[EI]} = \frac{[I][\text{state 12}]p_{30}}{[\text{state 30}]p_{12}} = [I]\frac{k_{\text{off,I,12}}}{k_{\text{on,12}}[I]}\frac{p_{30}}{p_{12}} = \frac{k_{\text{off,I}}p_{12}}{p_{12}+p_{15}}\frac{p_{30}+p_{33}}{k_{\text{on}}p_{30}}\frac{p_{30}}{p_{12}} = \frac{k_{\text{off,I}}}{k_{\text{on}}} \times \frac{p_{30}+p_{33}}{p_{12}+p_{15}}$$

The sum, $p_{30} + p_{33}$, is the combined population of the binding competent conformations in the EI complex, whereas $p_{12} + p_{15}$ is the combined population of the binding competent conformations in free enzyme E. The rates $k_{off,I}$ and k_{on} are part of the global parameters that are obtained in the least-squares fit and these apply to wild-type and mutants of HDAC8. Thus, the difference in K_d between wild-type and mutants of HDAC8 is given by the different populations of the binding competent states in free enzyme and bound enzyme, which in turn are derived from the CPMG relaxation dispersion data.

Supplementary figures:

Fig. S1.

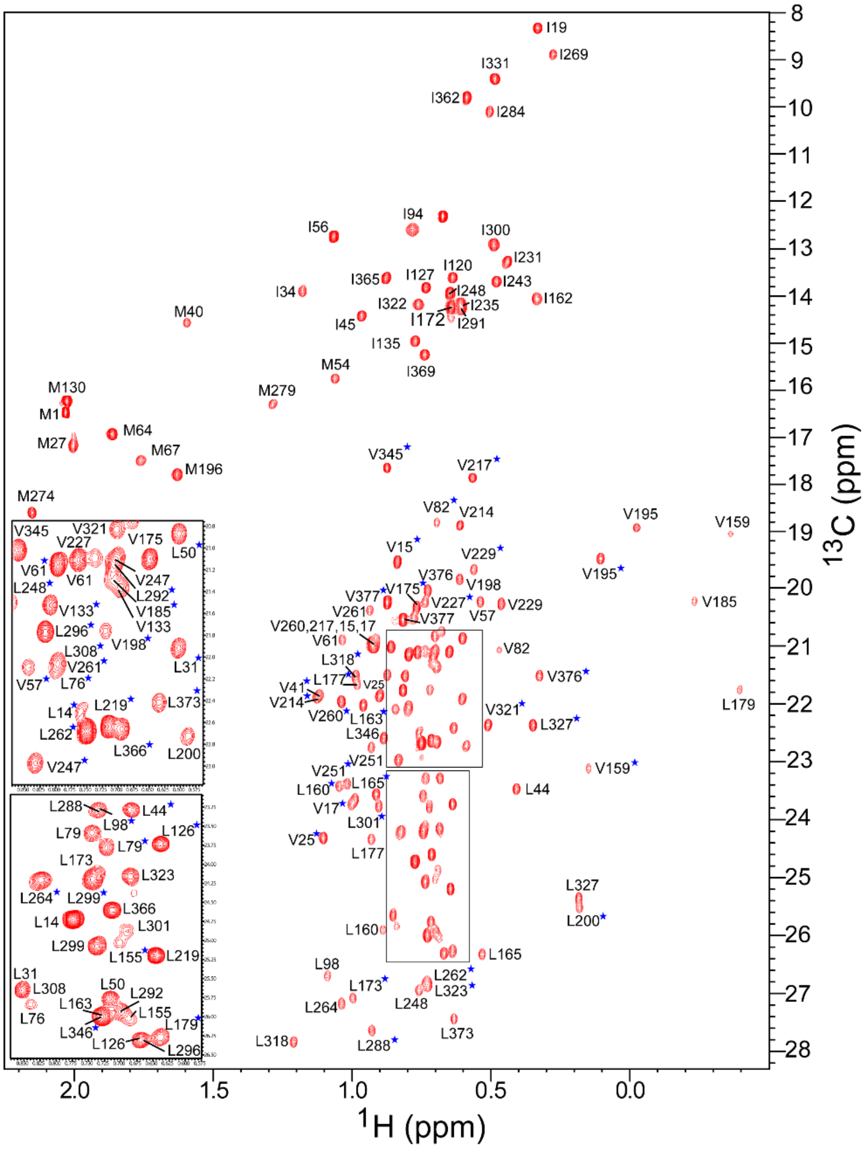

Fig. S1. Chemical shift assignment of I, L, V, and M methyl groups in HDAC8. Assigned methyl-TROSY spectrum of free HDAC8 acquired on an ILVM specifically labelled sample (see Material and Methods) at a static magnetic field strength of 22.3 T. The assignment for methyl groups of 23 out of the 25 Ile (I108 and I115 remain unassigned), 26 Val, 32 Leu, and 10 Met were obtained. Star symbols in the figure represent the assignment of $^{13}C^{\delta2}/^{1}H^{\delta2}$ and $C^{\gamma2}/H^{\gamma2}$ for Leu and Val residue, respectively, which were obtained though stereospecific assignment.

Fig. S2.

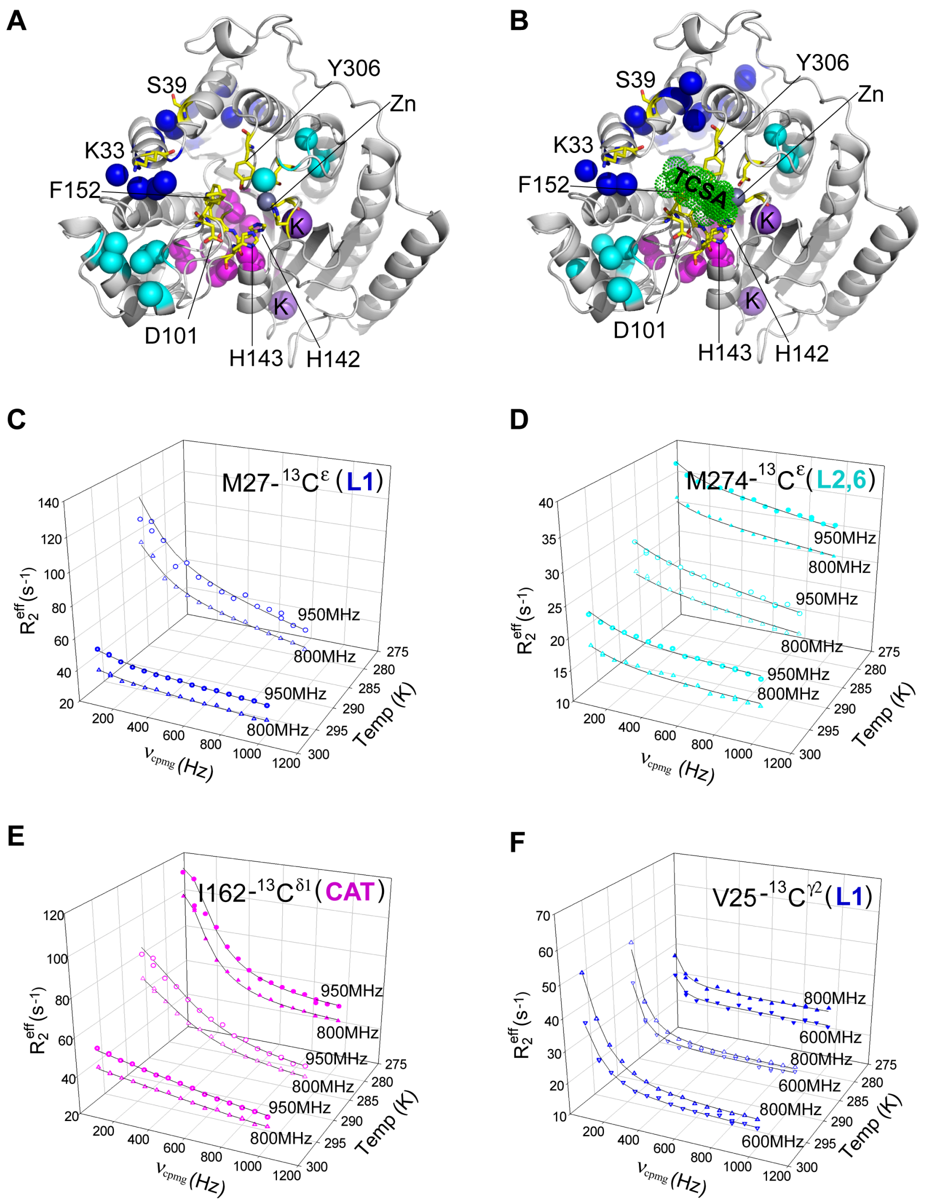

Fig. S2. Residues from L1, L2, L6, and the catalytic regions show dispersion in both free HDAC8 and HDAC8:TCSA. (**A**) and (**B**) highlight the methyl groups showing dispersion in free HDAC8 and HDAC8:TCSA, respectively. Methyl groups of HDAC8 indicating the presence of one and two sparsely-populated conformational states in the MQ-CPMG experiment are shown in magenta and blue colour, respectively. In (**A**) and (**B**), residues critical for deacetylation activity and enzymatic regulation are shown as yellow sticks. (**C**), (**D**), (**E**), and (**F**) show 3D plot of relaxation dispersion profiles for M27, I162, and M274 in free HDAC8, and V25$^{C\gamma 2/H\gamma 2}$ in the HDAC8:TCSA complex, respectively, as a function of temperature. In (**C**), (**D**), (**E**), and (**F**) data acquired at magnetic field strengths of 22.3, 18.8, and 14.1 T are shown as tringle up (▲), sphere (●), and tringle down (▼), respectively; and data acquired at 298K, 288K, and 278K are shown as closed symbols, open symbols, and bold open symbols.

Fig. S3.

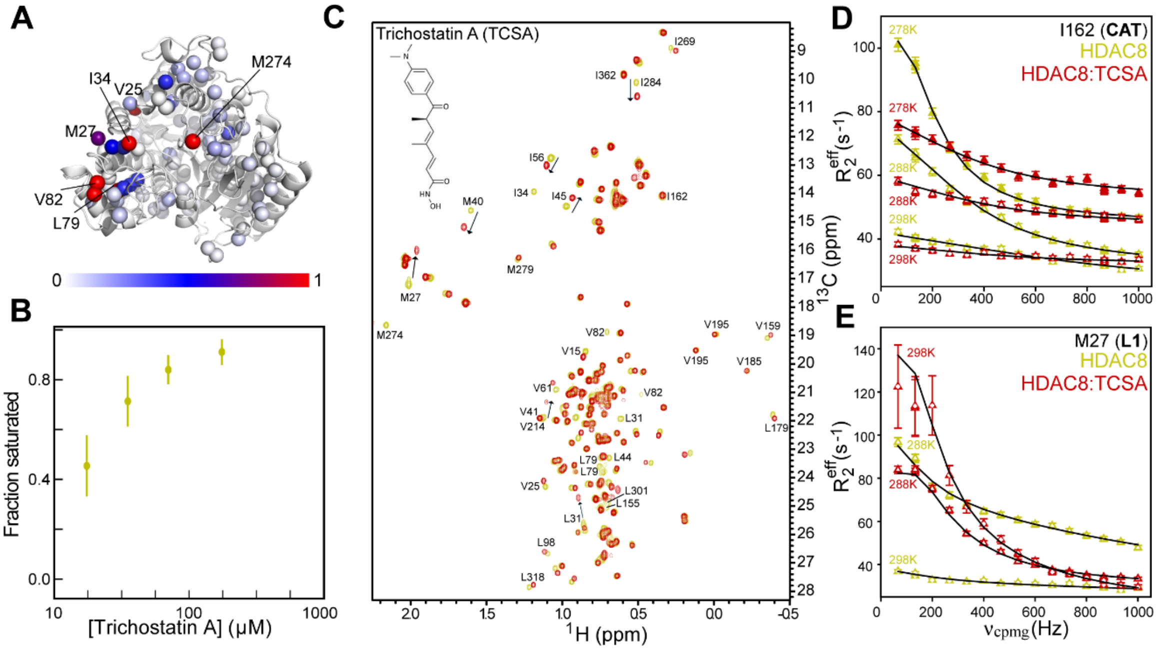

Fig. S3. Effect of Inhibitor binding on HDAC8. (**A**) shows structural representation chemical shift changes, ΔCS, upon TCSA binding. The chemical shift changes (ΔCS) upon trichostatin A binding were calculated as $\Delta CS_{\mathrm{I,L,V,M}} = \sqrt{(\Delta\delta_{\mathrm{H}}/\alpha)^2 + (\Delta\delta_{\mathrm{C}}/\beta)^2}$, where α and β are the standard deviations of the ^{1}H and ^{13}C chemical shifts for methyl groups, deposited in the Biological Magnetic Resonance Data Bank (3). Residues I34, L79, V82, and M274 are coloured red due to severe peak broadening upon saturation with the inhibitor. (**B**) shows fraction of saturated HDAC8:TCSA complex as a function of the concentration of the inhibitor trichostatin A. Each data point in the plot represents the average of the ratio of the populations of the free and bound state for five residues I45, I56, I269, I284, and I331, measured from the intensities of the individual peaks. Error for each data point represent the standard deviation (s.d.) calculated from the population for the five residues for each titration point. (**C**) shows overlay of methyl-TROSY NMR spectra of free HDAC8 (yellow) and HDAC8 with 2.5 equivalent inhibitor trichostatin A (red). Distinct changes are observed for the labelled residues and change in position of the cross peaks upon inhibitor binding are marked with an arrow. (**D**) and (**E**) show methyl MQ-CPMG relaxation dispersion profiles of I162 and M27, respectively, for free HDAC8 (yellow) and in the HDAC8:TCSA complex (red) at 18.8 T. In (**D**) and (**E**) data acquired at 278K, 288K, and 298K are shown in closed tringle, open tringle, and x-hair tringle, respectively.

Fig. S4.

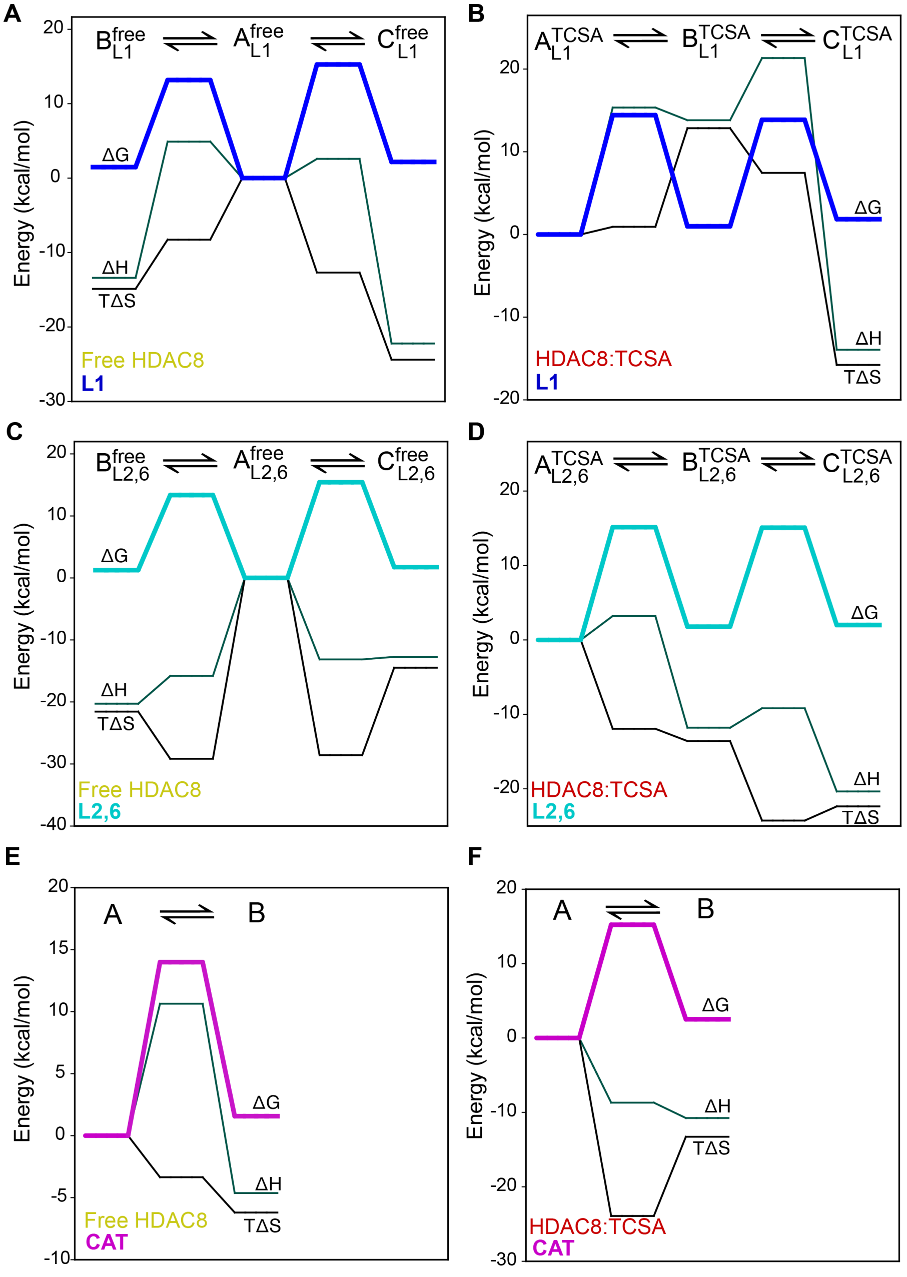

Fig. S4. Energy diagrams for free HDAC8 and HDAC8:TCSA at 298K. (**A**) and (**B**) show Gibbs free-energy levels of the majorly populated ground state and sparsely-populated states for the L1 region for free HDAC8 (A^{free}, B^{free}, C^{free}) and in the HDAC8:TCSA complex (A^{TCSA}, B^{TCSA}, C^{TCSA}), respectively. (**C**) and (**D**) show energy levels of the sampled states in the L2,6 region for free HDAC8 and HDAC8:TCSA, respectively. (**E**) and (**F**) show energy levels of the A and B states for the catalytic region (CAT) in free HDAC8 and in HDAC8:TCSA, respectively.

Fig. S5.

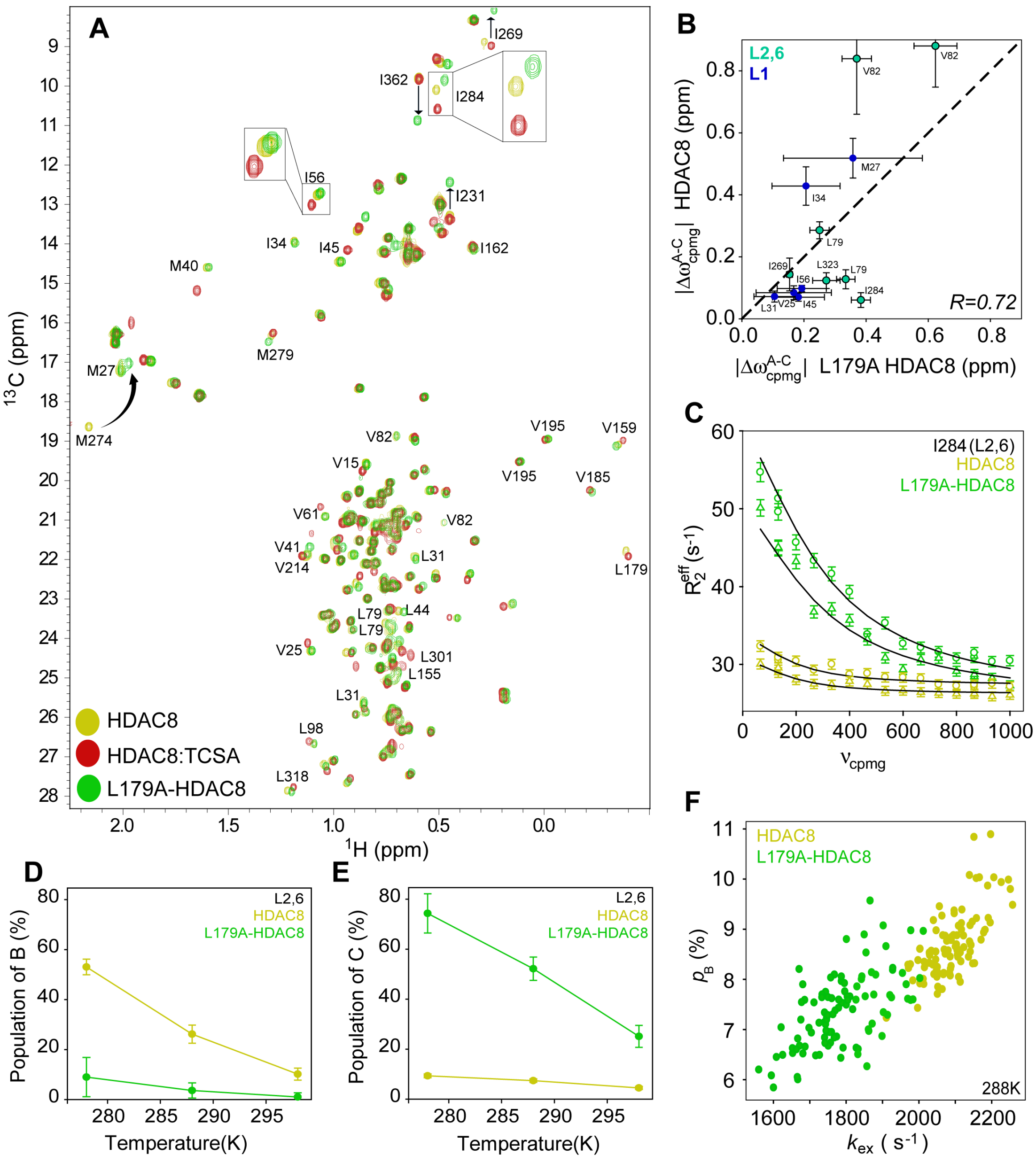

Fig. S5. Effect of L179A mutation. (**A**) Overlay of methyl-TROSY NMR spectra of free HDAC8 (yellow), HDAC8 with 2.5eq inhibitor (red), and L179A-HDAC8 (green). (**B**) Correlation plot between |Δω| obtained from CPMG relaxation dispersion experiments for the E_2 state of L1 and L2,6 region in wild-type HDAC8 and in L179A-HDAC8, *R* is the Pearson correlation coefficient. (**C**) Methyl MQ-CPMG relaxation dispersion profiles of I284. Wild-type HDAC8 and M274A-HDAC8 data acquired at 288K are shown in yellow and magenta colours, respectively. Data acquired at magnetic field strengths of 22.3 T and 18.8 T are shown with circles and tringle, respectively. (**D**) and (**E**) show the change in population of the “bound-state” (A^{TCSA} in HDAC8:TCSA), E_1, and the E_2 state, respectively, in wild-type HDAC8 and in L179A-HDAC8, as a function of temperature. (**F**) Bootstrap of exchange rate, k_{ex}, and population, p_B, from analysis of NMR MQ-CPMG data using a two-state exchange model for the catalytic region of wild-type HDAC8 and L179A-HDAC8 at 298K and 288K.

Fig. S6.

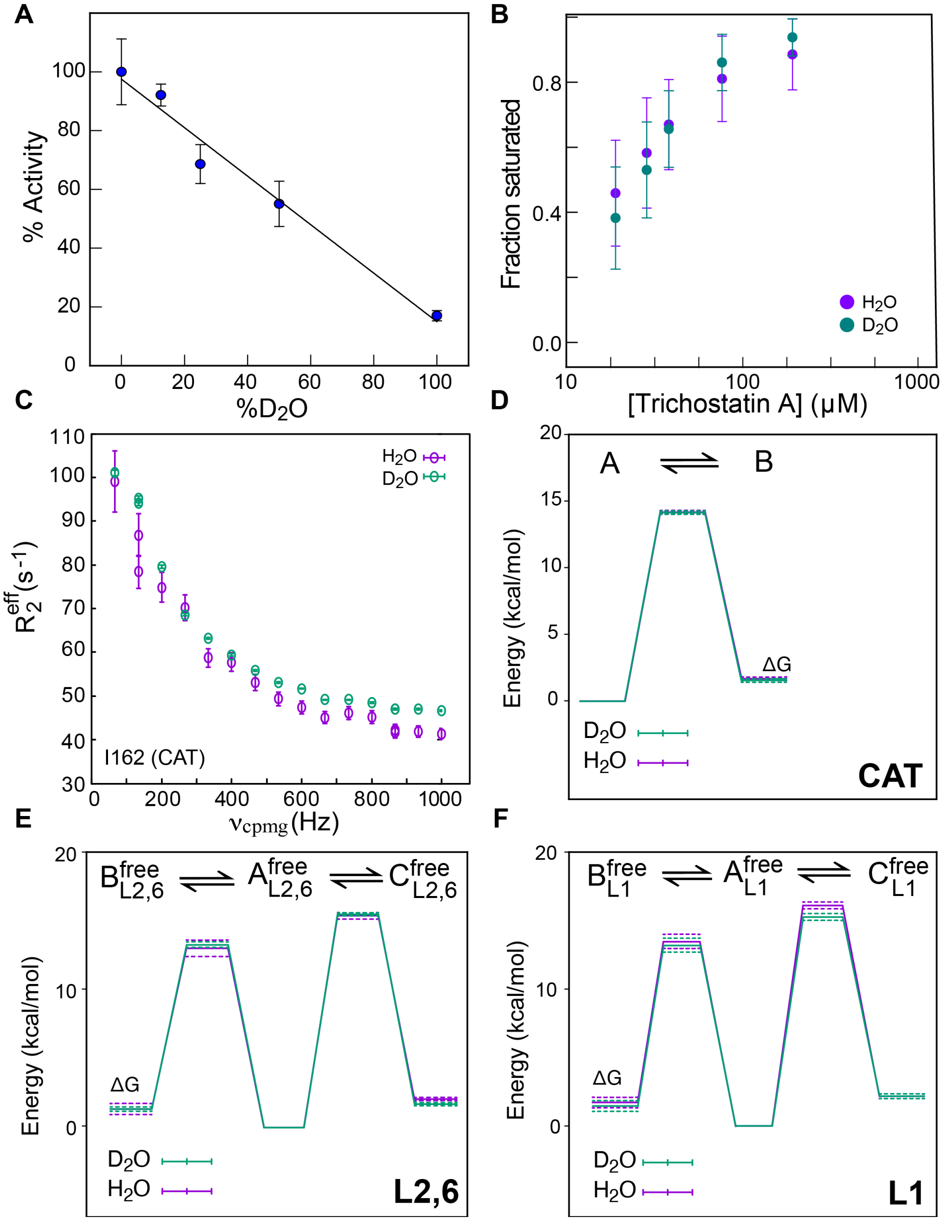

Fig. S6. Deuterium kinetic isotope effect. (**A**) Enzymatic activity as a function of the molar fraction of D_2O, where it is seen that HDAC8 is 6.2 times more active in H_2O compared to D_2O, thus $k_{cat}/K_M(D_2O) = 6.2 \pm 0.3 \times k_{cat}/K_M$ (D_2O). (**B**) Binding of the inhibitor, trochostatin A, to HDAC8 in H_2O and D_2O. (**C**) Representative MQ-CPMG relaxation dispersion profile of I162, (278 K, 18.8 T), showing minimal difference between experiments performed in H_2O and D_2O. (**D-F**) Comparisons of the free energy landscapes obtained from MQ-CPMG experiments performed in H_2O and D_2O for the three regions, CAT, L1, and L2,6. No significant changes are observed between H_2O and D_2O.

Fig. S7.

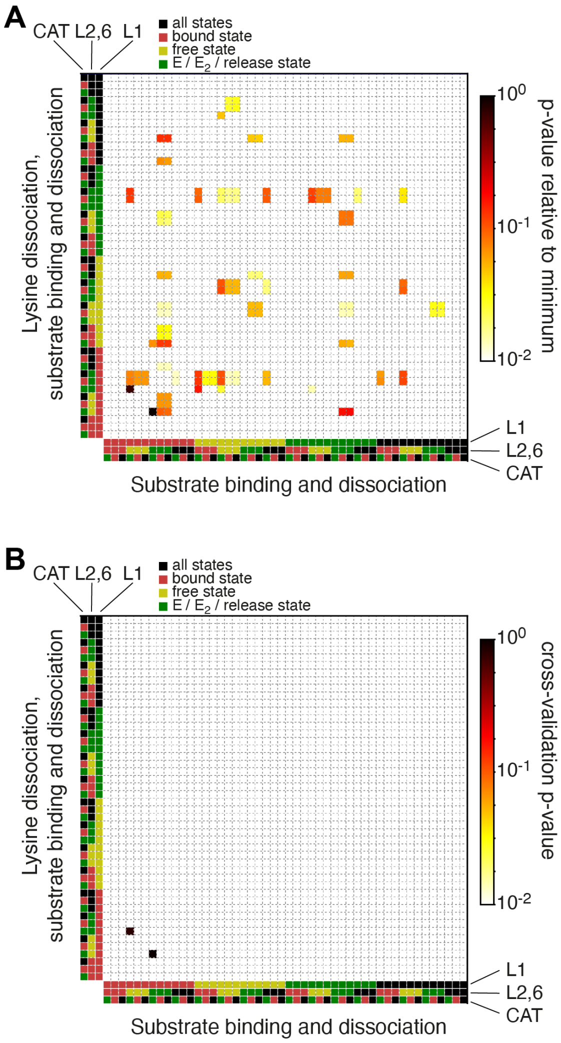

Fig. S7. Summary of least-squares fit and selection of best-fit model. (**A**) p-value compared with best-fit model, calculated using the model number as a discrete parameter. (**B**) p-value compared with best-fit model, including the cross-validation data from the M274A mutant of HDAC8. Here the χ^2 used to calculate the p-values are, $\chi^2_{fit} + \chi^2_{cross}$. The second-best model has a cross-validation p-value of 0.8 and all other models have cross-validation p-value less than 0.01.

Fig. S8.

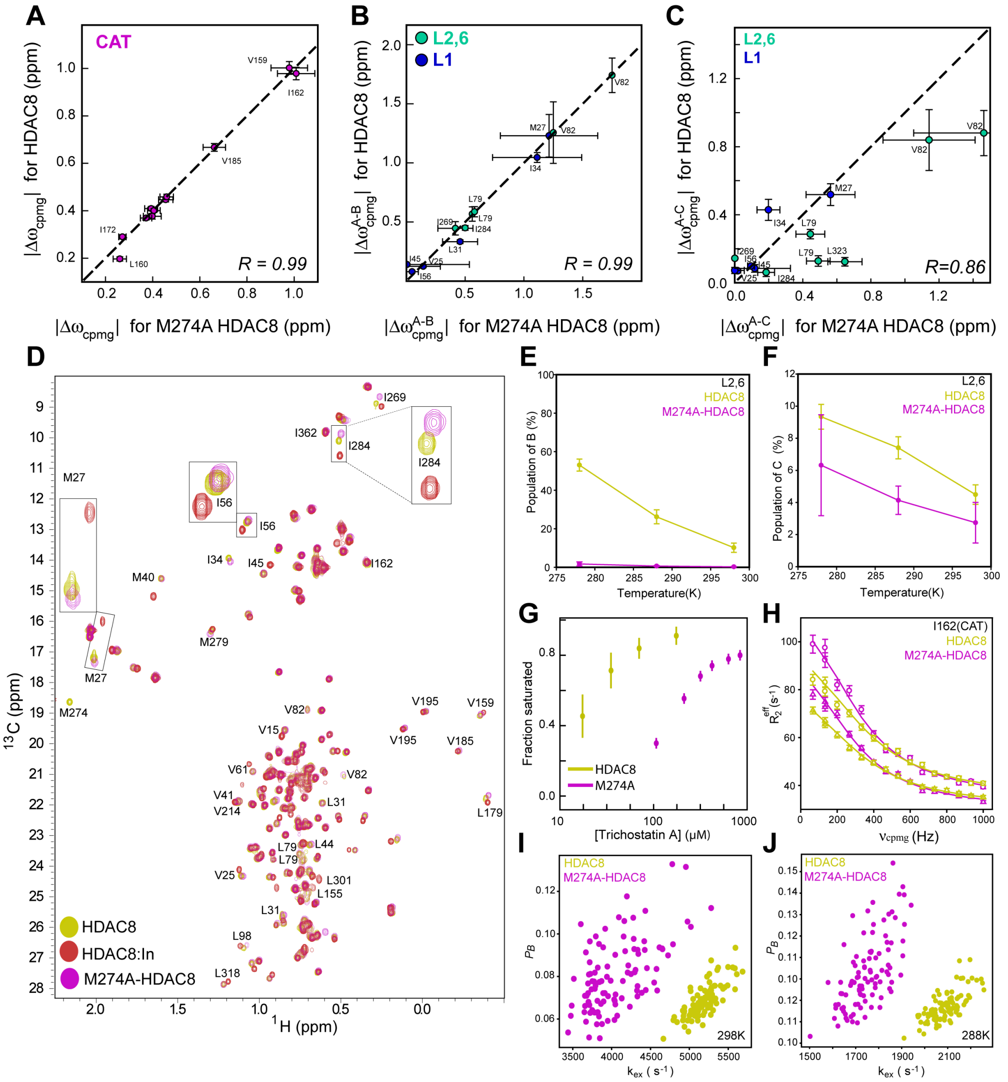

Fig. S8. Effect of M274A mutation. (**A-C**) Correlation plot between |Δω| obtained from MQ-CPMG relaxation dispersion experiments for the catalytic site low-populated state (**A**), the E_1 state (**B**), and the E_2 state of L1 and L2,6 region (**C**) in wild-type HDAC8 and in M274A-HDAC8, respectively. (**D**) Overlay of methyl-TROSY NMR spectra of free HDAC8 (yellow), HDAC8 with 2.5eq inhibitor (red), and the M274A-HDAC8 (magenta). (**E**) and (**F**) Change in population of "bound-conformation", E_1, and of E_2 states, respectively, in wild-type HDAC8 and in M274A-HDAC8, as a function of temperature. (**G**) An NMR titration of wild type HDAC8 and M274A-HDAC8 with trichostatin A inhibitor. Each data point in the plot represents the average of the ratio of populations of the free and bound state for five residues I45, I56, I269, I284, and I331. A standard deviation (vertical bar) for each titration point was calculated from the populations of the five residues. (**H**) Methyl MQ-CPMG relaxation dispersion profiles of I162. Wild-type HDAC8 and M274A-HDAC8 data, acquired at 288K, are shown in yellow and magenta colours, respectively. Data acquired at magnetic field strengths of 22.3 T and 18.8 T are shown in circles and tringles, respectively. (**I**) and (**J**) Bootstrap values of exchange rate, k_{ex}, and population, p_E, from fitting of MQ-CPMG data using a two-state exchange model for catalytic region in wild-type HDAC8 and in M274A-HDAC8, respectively.

Supplementary tables:

Table S1. Assigned ^1H and ^{13}C chemical shifts for the methyl groups of Ile (δ_1 only), Leu, Val, and Met of HDAC8.

	Free HDAC8		HDAC8:TCSA		L179A-HDAC8		M274A-HDAC8	
Assignment	^{13}C	^1H	^{13}C	^1H	^{13}C	^1H	^{13}C	^1H
$I120^{C\delta1/H\delta1}$	13.63	0.64	13.60	0.64	13.60	0.64	13.62	0.64
$I127^{C\delta1/H\delta1}$	13.84	0.73	13.83	0.74	13.83	0.73	13.84	0.73
$I135^{C\delta1/H\delta1}$	14.98	0.77	14.98	0.76	14.98	0.76	14.98	0.77
$I162^{C\delta1/H\delta1}$	14.08	0.34	14.05	0.34	14.12	0.33	14.07	0.34
$I172^{C\delta1/H\delta1}$	14.26	0.64	14.28	0.65	14.26	0.64	14.26	0.64
$I19^{C\delta1/H\delta1}$	8.33	0.33	8.33	0.33	8.30	0.33	8.33	0.33
$I231^{C\delta1/H\delta1}$	13.29	0.44	13.35	0.45	12.45	0.45	13.27	0.44
$I235^{C\delta1/H\delta1}$	14.18	0.61	14.18	0.61	14.18	0.61	14.20	0.61
$I243^{C\delta1/H\delta1}$	13.71	0.48	13.70	0.48	13.60	0.50	13.70	0.48
$I269^{C\delta1/H\delta1}$	8.88	0.28	8.96	0.25	8.08	0.24	8.68	0.26
$I284^{C\delta1/H\delta1}$	10.10	0.50	10.57	0.51	9.84	0.47	9.90	0.49
$I291^{C\delta1/H\delta1}$	14.28	0.61	14.28	0.61	14.28	0.61	14.28	0.61
$I300^{C\delta1/H\delta1}$	12.92	0.49	12.98	0.49	13.02	0.49	12.91	0.49
$I322^{C\delta1/H\delta1}$	14.20	0.76	14.21	0.76	14.17	0.76	14.19	0.76
$I331^{C\delta1/H\delta1}$	9.40	0.49	9.29	0.51	9.45	0.46	9.44	0.47
$I34^{C\delta1/H\delta1}$	13.93	1.18	***	***	13.97	1.18	14.06	1.17
$I348^{C\delta1/H\delta1}$	13.96	0.65	14.07	0.64	14.03	0.70	14.02	0.65
$I362^{C\delta1/H\delta1}$	9.80	0.59	9.81	0.59	10.86	0.60	9.80	0.59
$I365^{C\delta1/H\delta1}$	13.64	0.88	13.58	0.88	13.32	0.85	13.64	0.88
$I369^{C\delta1/H\delta1}$	15.27	0.74	15.28	0.75	15.15	0.74	15.27	0.74
$I45^{C\delta1/H\delta1}$	14.44	0.97	14.14	0.93	14.44	0.96	14.45	0.97
$I56^{C\delta1/H\delta1}$	12.76	1.07	12.98	1.10	12.71	1.06	12.72	1.06
$I94^{C\delta1/H\delta1}$	12.61	0.78	12.48	0.79	12.60	0.78	12.64	0.77
$L126^{C\delta1/H\delta1}$	26.28	0.64	26.22	0.64	26.28	0.64	26.28	0.64
$L126^{C\delta2/H\delta2}$	23.73	0.64	23.66	0.64	23.72	0.64	23.74	0.64
$L14^{C\delta1/H\delta1}$	24.73	0.77	24.74	0.78	24.71	0.77	22.50	0.76
$L14^{C\delta2/H\delta2}$	22.50	0.76	22.53	0.76	22.45	0.76	24.73	0.77
$L155^{C\delta1/H\delta1}$	26.08	0.68	26.08	0.68	25.03	0.70	26.08	0.68
$L155^{C\delta2/H\delta2}$	25.03	0.70	24.39	0.64	26.04	0.69	25.03	0.70
$L160^{C\delta1/H\delta1}$	25.90	0.89	25.90	0.90	25.92	0.89	23.39	1.05
$L160^{C\delta2/H\delta2}$	23.42	1.05	23.38	1.05	23.41	1.04	25.88	0.89
$L163^{C\delta1/H\delta1}$	25.99	0.73	25.94	0.73	25.96	0.72	22.03	0.96
$L163^{C\delta2/H\delta2}$	22.03	0.96	22.00	0.97	22.01	0.96	25.99	0.73
$L165^{C\delta1/H\delta1}$	26.33	0.53	26.36	0.54	23.53	0.91	23.56	0.91

L165 $^{C\delta2/H\delta2}$	23.56	0.91	23.53	0.92	26.33	0.53	26.31	0.53
L173 $^{C\delta1/H\delta1}$	24.14	0.74	24.09	0.74	24.03	0.73	27.09	1.00
L173 $^{C\delta2/H\delta2}$	27.09	1.00	27.08	1.00	27.06	0.99	24.14	0.74
L177 $^{C\delta1/H\delta1}$	24.34	0.93	24.34	0.92	24.30	0.91	21.68	0.98
L177 $^{C\delta2/H\delta2}$	21.66	0.98	21.73	0.98	21.68	0.96	24.34	0.93
L179 $^{C\delta1/H\delta1}$	21.76	-0.40	21.89	-0.40			21.70	-0.41
L179 $^{C\delta2/H\delta2}$	26.18	0.64	26.23	0.64			26.15	0.59
L200 $^{C\delta1/H\delta1}$	22.73	0.59	22.72	0.59	22.67	0.58	25.51	0.18
L200 $^{C\delta2/H\delta2}$	25.51	0.18	25.50	0.19	25.55	0.18	22.73	0.59
L219 $^{C\delta1/H\delta1}$	25.20	0.65	25.20	0.66	25.17	0.65	25.20	0.65
L219 $^{C\delta2/H\delta2}$	22.64	0.72	22.66	0.73	22.64	0.72	22.64	0.72
L248 $^{C\delta1/H\delta1}$	26.95	0.76	26.96	0.76	26.91	0.76	26.95	0.76
L248 $^{C\delta2/H\delta2}$	21.50	0.87	21.49	0.88	21.54	0.87	21.50	0.87
L262 $^{C\delta1/H\delta1}$	26.84	0.73	26.72	0.72	22.68	0.75	26.84	0.73
L262 $^{C\delta2/H\delta2}$	22.69	0.75	22.68	0.76	26.87	0.72	22.69	0.75
L264 $^{C\delta1/H\delta1}$	27.18	1.04	27.31	1.03	24.25	0.81	24.20	0.82
L264 $^{C\delta2/H\delta2}$	24.21	0.82	24.20	0.82	27.23	1.04	27.15	1.04
L288 $^{C\delta1/H\delta1}$	23.29	0.74	23.23	0.73	27.60	0.93	27.68	0.93
L288 $^{C\delta2/H\delta2}$	27.64	0.93	27.53	0.92	23.27	0.74	23.32	0.74
L292 $^{C\delta1/H\delta1}$	25.94	0.70	25.89	0.71	25.83	0.70	25.94	0.70
L292 $^{C\delta2/H\delta2}$	21.15	0.71	21.20	0.71	21.15	0.71	21.16	0.71
L296 $^{C\delta1/H\delta1}$	26.31	0.67	26.31	0.68	26.30	0.67	21.77	0.82
L296 $^{C\delta2/H\delta2}$	21.77	0.82	21.76	0.82	21.77	0.82	26.31	0.67
L299 $^{C\delta1/H\delta1}$	25.07	0.74	25.10	0.75	25.04	0.74	24.22	0.74
L299 $^{C\delta2/H\delta2}$	24.20	0.74	24.18	0.75	24.22	0.74	25.06	0.74
L301 $^{C\delta1/H\delta1}$	24.88	0.69	24.68	0.68	24.84	0.70	24.88	0.70
L301 $^{C\delta2/H\delta2}$	23.77	0.90	23.76	0.91	23.77	0.90	23.77	0.90
L308 $^{C\delta1/H\delta1}$	25.65	0.85	25.75	0.85	21.96	0.80	25.61	0.85
L308 $^{C\delta2/H\delta2}$	21.96	0.80	21.96	0.80	25.62	0.85	21.96	0.80
L31 $^{C\delta1/H\delta1}$	25.50	0.85	24.69	0.89	25.50	0.85	25.50	0.85
L31 $^{C\delta2/H\delta2}$	21.92	0.60	21.89	0.60	21.98	0.61	21.92	0.60
L318 $^{C\delta1/H\delta1}$	27.84	1.21	27.73	1.19	27.86	1.20	21.52	0.99
L318 $^{C\delta2/H\delta2}$	21.53	0.99	21.45	0.97	21.49	0.98	27.87	1.21
L323 $^{C\delta1/H\delta1}$	24.16	0.69	24.30	0.68	24.06	0.69	24.14	0.69
L323 $^{C\delta2/H\delta2}$	26.83	0.74	26.72	0.72	26.80	0.73	26.86	0.73
L327 $^{C\delta1/H\delta1}$	25.36	0.18	25.34	0.20	22.34	0.35	22.36	0.35
L327 $^{C\delta2/H\delta2}$	22.37	0.35	22.48	0.36	25.36	0.18	25.35	0.18
L346 $^{C\delta1/H\delta1}$	22.59	0.89	22.39	0.88	22.67	0.88	22.61	0.89
L346 $^{C\delta2/H\delta2}$	26.04	0.73	26.04	0.73	26.04	0.73	26.04	0.73
L366 $^{C\delta1/H\delta1}$	24.60	0.71	24.63	0.72	22.70	0.70	22.66	0.69
L366 $^{C\delta2/H\delta2}$	22.66	0.69	22.62	0.70	24.48	0.72	24.61	0.71

L373 $^{Cδ1/Hδ1}$	27.43	0.63	27.42	0.64	27.40	0.63	22.42	0.63
L373 $^{Cδ2/Hδ2}$	22.42	0.63	22.40	0.64	22.39	0.63	27.43	0.63
L44 $^{Cδ1/Hδ1}$	23.47	0.41	23.43	0.45	23.32	0.66	23.48	0.41
L44 $^{Cδ2/Hδ2}$	23.29	0.68	23.29	0.72	23.47	0.41	23.29	0.67
L50 $^{Cδ1/Hδ1}$	25.77	0.72	25.85	0.69	25.79	0.72	25.77	0.72
L50 $^{Cδ2/Hδ2}$	20.87	0.60	20.91	0.60	20.92	0.60	20.88	0.60
L76 $^{Cδ1/Hδ1}$	25.84	0.84	25.75	0.85	22.11	0.80	25.84	0.85
L76 $^{Cδ2/Hδ2}$	22.11	0.80	22.28	0.81	25.86	0.84	22.11	0.80
L79 $^{Cδ1/Hδ1}$	23.58	0.74	***	***	23.63	0.73	23.84	0.73
L79 $^{Cδ2/Hδ2}$	23.78	0.72	***	***	23.78	0.72	23.60	0.75
L98 $^{Cδ1/Hδ1}$	26.70	1.09	26.58	1.12	26.66	1.09	26.60	1.07
L98 $^{Cδ2/Hδ2}$	23.55	0.71	23.55	0.71	23.55	0.71	23.55	0.71
M1 $^{Cε/Hε}$	16.49	2.03	16.49	2.04	16.48	2.03	16.49	2.03
M130 $^{Cε/Hε}$	16.26	2.03	16.27	2.03	16.26	2.03	16.27	2.03
M196 $^{Cε/Hε}$	17.81	1.63	17.83	1.64	17.84	1.63	17.81	1.63
M27 $^{Cε/Hε}$	17.20	2.01	16.01	1.96	17.17	2.01	17.36	2.01
M274 $^{Cε/Hε}$	18.62	2.15	19.10	2.03	17.02	1.97		
M279 $^{Cε/Hε}$	16.32	1.29	16.23	1.29	16.48	1.31	16.42	1.30
M40 $^{Cε/Hε}$	14.59	1.60	15.15	1.65	14.59	1.59	14.63	1.59
M54 $^{Cε/Hε}$	15.77	1.06	15.83	1.06	15.79	1.06	15.77	1.06
M64 $^{Cε/Hε}$	16.96	1.86	16.92	1.90	16.96	1.86	16.96	1.87
M67 $^{Cε/Hε}$	17.52	1.76	17.52	1.75	17.49	1.75	17.51	1.77
V133 $^{Cγ1/Hγ1}$	21.37	0.70	21.44	0.70	21.33	0.69	21.37	0.70
V133 $^{Cγ2/Hγ2}$	21.53	0.81	21.57	0.81	21.53	0.81	21.53	0.81
V15 $^{Cγ1/Hγ1}$	20.93	0.92	20.93	0.92	19.59	0.84	20.93	0.92
V15 $^{Cγ2/Hγ2}$	19.55	0.84	19.73	0.86	20.93	0.92	19.56	0.84
V159 $^{Cγ1/Hγ1}$	19.07	-0.36	18.95	-0.37	19.11	-0.34	19.07	-0.36
V159 $^{Cγ2/Hγ2}$	23.11	0.15	23.15	0.19	23.11	0.15	23.09	0.14
V17 $^{Cγ1/Hγ1}$	20.91	0.91	20.91	0.91	23.71	1.00	23.73	1.00
V17 $^{Cγ2/Hγ2}$	23.73	1.00	23.68	1.00	20.91	0.91	20.91	0.91
V175 $^{Cγ1/Hγ1}$	20.76	0.68	20.80	0.68	20.79	0.67	20.52	0.78
V175 $^{Cγ2/Hγ2}$	20.52	0.78	20.54	0.78	20.52	0.78	20.79	0.67
V185 $^{Cγ1/Hγ1}$	20.23	-0.23	20.21	-0.22	21.30	0.71	21.34	0.71
V185 $^{Cγ2/Hγ2}$	21.30	0.71	21.26	0.74	20.28	-0.23	20.23	-0.23
V195 $^{Cγ1/Hγ1}$	18.94	-0.03	18.94	-0.01	19.50	0.11	19.49	0.11
V195 $^{Cγ2/Hγ2}$	19.49	0.11	19.49	0.12	18.93	-0.02	18.95	-0.03
V198 $^{Cγ1/Hγ1}$	19.85	0.61	19.84	0.62	21.70	0.72	19.85	0.61
V198 $^{Cγ2/Hγ2}$	21.76	0.72	21.74	0.72	19.81	0.61	21.76	0.72
V214 $^{Cγ1/Hγ1}$	18.90	0.61	18.90	0.61	21.68	1.11	21.88	1.12
V214 $^{Cγ2/Hγ2}$	21.88	1.12	21.88	1.15	18.97	0.61	18.92	0.61
V217 $^{Cγ1/Hγ1}$	21.00	0.93	21.04	0.96	21.00	0.93	21.00	0.93

V217 $^{C\gamma2/H\gamma2}$	17.88	0.57	17.86	0.57	17.86	0.56	17.88	0.57
V227 $^{C\gamma1/H\gamma1}$	21.09	0.74	21.09	0.74	21.09	0.74	20.25	0.74
V227 $^{C\gamma2/H\gamma2}$	20.25	0.74	20.25	0.74	20.25	0.73	21.09	0.74
V229 $^{C\gamma1/H\gamma1}$	20.28	0.46	20.23	0.46	20.33	0.46	20.26	0.46
V229 $^{C\gamma2/H\gamma2}$	19.69	0.56	19.67	0.57	19.73	0.56	19.70	0.56
V247 $^{C\gamma1/H\gamma1}$	21.12	0.70	21.03	0.71	21.12	0.70	22.98	0.83
V247 $^{C\gamma2/H\gamma2}$	22.98	0.83	22.95	0.84	22.99	0.84	21.05	0.70
V25 $^{C\gamma1/H\gamma1}$	21.87	0.90	21.87	0.90	24.31	1.10	24.34	1.09
V25 $^{C\gamma2/H\gamma2}$	24.31	1.10	24.09	1.12	21.86	0.90	21.87	0.90
V251 $^{C\gamma1/H\gamma1}$	22.75	0.93	22.75	0.93	22.75	0.93	22.75	0.93
V251 $^{C\gamma2/H\gamma2}$	23.38	1.02	23.35	1.02	23.35	1.02	23.38	1.02
V260 $^{C\gamma1/H\gamma1}$	21.04	0.93	21.00	0.93	21.04	0.93	21.04	0.93
V260 $^{C\gamma2/H\gamma2}$	21.97	1.04	21.96	1.04	21.97	1.04	21.97	1.04
V261 $^{C\gamma1/H\gamma1}$	20.39	0.94	20.41	0.94	20.36	0.93	22.08	0.80
V261 $^{C\gamma2/H\gamma2}$	22.08	0.80	22.07	0.80	22.08	0.80	20.40	0.94
V282 $^{C\gamma1/H\gamma1}$	20.34	0.77	20.34	0.77	20.32	0.77	20.34	0.77
V282 $^{C\gamma2/H\gamma2}$	21.10	0.65	21.10	0.65	21.00	0.64	21.10	0.65
V321 $^{C\gamma1/H\gamma1}$	20.83	0.70	20.82	0.70	20.82	0.70	20.83	0.70
V321 $^{C\gamma2/H\gamma2}$	22.37	0.51	22.44	0.51	22.45	0.52	22.37	0.51
V345 $^{C\gamma1/H\gamma1}$	21.02	0.86	20.99	0.87	17.65	0.87	21.03	0.86
V345 $^{C\gamma2/H\gamma2}$	17.67	0.88	17.62	0.88	21.02	0.86	17.67	0.87
V376 $^{C\gamma1/H\gamma1}$	20.04	0.73	20.04	0.73	21.49	0.33	21.51	0.32
V376 $^{C\gamma2/H\gamma2}$	21.51	0.33	21.51	0.33	20.04	0.73	20.05	0.73
V377 $^{C\gamma1/H\gamma1}$	20.55	0.82	20.55	0.82	20.54	0.82	20.25	0.87
V377 $^{C\gamma2/H\gamma2}$	20.24	0.87	20.26	0.88	20.24	0.88	20.55	0.82
V41 $^{C\gamma1/H\gamma1}$	20.87	1.04	20.65	1.06	20.90	1.04	20.92	1.03
V41 $^{C\gamma2/H\gamma2}$	21.84	1.12	21.34	1.10	21.87	1.12	21.84	1.12
V57 $^{C\gamma1/H\gamma1}$	20.24	0.54	20.21	0.52	20.23	0.54	20.24	0.54
V57 $^{C\gamma2/H\gamma2}$	22.09	0.84	22.09	0.84	22.09	0.84	22.09	0.84
V61 $^{C\gamma1/H\gamma1}$	21.11	0.76	21.18	0.77	21.10	0.79	21.08	0.76
V61 $^{C\gamma2/H\gamma2}$	21.15	0.80	21.29	0.81	21.12	0.76	21.17	0.80
V82 $^{C\gamma1/H\gamma1}$	21.09	0.47	***	***	21.05	0.47	18.91	0.70
V82 $^{C\gamma2/H\gamma2}$	18.83	0.70	***	***	18.86	0.70	21.03	0.48

*** Represent resonances, which are broadened beyond detection upon addition of the inhibitor TCSA.

Table S2. Reduced chi-squared obtained for analysis of MQ-CPMG data with different models[a)]

	**Two-site exchange**		
	Region	A ⇌ B	
Free HDAC8	L1	0.73	
	L2,6	0.93	
	CAT	**0.67**[a)]	
HDAC8:TCSA	L1	2.5	
	L2,6	2.0	
	CAT	**1.27**[a)]	
	**Three-site exchange**		
	Region	(bifurcated) B ⇌ A ⇌ C	(linear) A ⇌ B ⇌ C / A ⇌ C ⇌ B
Free HDAC8	L1	**0.35**[a)]	0.35
	L2,6	**0.74**[a)]	0.81
	CAT	0.61	0.65
HDAC8:TCSA	L1	0.86	**0.82**[a)]
	L2,6	0.45	**0.42**[a)]
	CAT	1.15	1.42

[a)] The models chosen are highlighted in bold.

Table S3. Analysis of MQ-CPMG

Panel A: Residues of HDAC8 with significant dispersions and used in the analysis

Regions	Residues with significant relaxation dispersion in free HDAC8[a)]	Residues showing relaxation dispersion in HDAC8:TCSA [a)]
L1	V25, M27, L31, I34, I45, I56, L323	V15, V17, V25, M27, L31, M40, V41, I45, M54, I56, L323, L327
L2,6	(L2): L79, V82, I94, L98<br>(L6): I269, M274, I284	(L2): M64, M67, I94, L98<br>(L6): I269, I284
CAT	I162, V159, L160, L165, I172, V185, V261, L299, L301	I162, L165, V159, V185, V261

[a)] Defined as residues with $R_{ex} = R_2^{eff}(30\ Hz) - R_2^{eff}(1000\ Hz) > 5\ s^{-1}$

Panel B: Populations and rates determined

Free HDAC8 (in H_2O)

	L1	L2,6	CAT
p(B) 298K (%)	4.9 ± 2.9	9.2 ± 7.0	5.2 ± 0.5
p(B) 288K (%)	18.0 ± 5.3	24.5 ± 11.9	7.0 ± 0.3
p(B) 278K (%)	45.3 ± 6.8	51.8 ± 12.2	9.5 ± 0.4
p(C) 298K (%)	2.3 ± 0.6	2.7 ± 0.8	
p(C) 288K (%)	7.9 ± 2.5	4.7 ± 0.9	
p(C) 278K (%)	18.4 ± 10	6.2 ± 1.7	
k_{ex}^{A-B} 298K (s^{-1})	16748 ± 5690	17427 ± 4584	5996 ± 451
k_{ex}^{A-B} 288K (s^{-1})	10166 ± 1570	14923 ± 4193	4248 ± 122
k_{ex}^{A-B} 278K (s^{-1})	9050 ± 2059	17046 ± 5873	1077 ± 57
k_{ex}^{A-C} 298K (s^{-1})	412 ± 223	1004 ± 347	
k_{ex}^{A-C} 288K (s^{-1})	272 ± 135	817 ± 167	
k_{ex}^{A-C} 278K (s^{-1})	220 ± 137	683 ± 269	

Free HDAC8 (in D_2O)

	L1	L2,6	CAT
p(B) 298K (%)	7.4 ± 4.7	10.2 ± 2.4	6.6 ± 0.5
p(B) 288K (%)	14.2 ± 4.2	26.2 ± 3.6	8.5 ± 0.4
p(B) 278K (%)	23.4 ± 4.2	53.0 ± 3.1	11.0 ± 0.4
p(C) 298K (%)	2.3 ± 0.4	4.5 ± 0.6	
p(C) 288K (%)	7.4 ± 0.7	7.4 ± 0.7	
p(C) 278K (%)	21.2 ± 5.6	9.3 ± 0.8	
k_{ex}^{A-B} 298K (s^{-1})	17927 ± 5229	9327 ± 845	5142 ± 143
k_{ex}^{A-B} 288K (s^{-1})	6430 ± 1072	8616 ± 417	2071 ± 49
k_{ex}^{A-B} 278K (s^{-1})	2362 ± 281	10845 ± 1026	788 ± 56
k_{ex}^{A-C} 298K (s^{-1})	1593 ± 204	587 ± 117	
k_{ex}^{A-C} 288K (s^{-1})	380 ± 98	613 ± 70	
k_{ex}^{A-C} 278K (s^{-1})	97 ± 27	681 ± 72	

L179A-HDAC8 (in D_2O)

	L1	L2,6	CAT
p(B) 298K (%)	2.2 ± 0.6	1.0 ± 1.7	5.2 ± 0.7

p(B) 288K (%)	8.3 ± 0.5	3.6 ± 3.0	7.3 ± 0.7
p(B) 278K (%)	27.0 ± 5.3	9.0 ± 7.8	10.3 ± 0.7
p(C) 298K (%)	2.5 ± 1.9	25.1 ± 4.4	
p(C) 288K (%)	5.4 ± 3.5	52.2 ± 4.7	
p(C) 278K (%)	10.0 ± 10.2	74.3 ± 7.8	
k_{ex}^{A-B} 298K (s^{-1})	5164 ± 1116	101360 ± 139481	4857 ± 222
k_{ex}^{A-B} 288K (s^{-1})	2575 ± 304	8221 ± 5380	1802 ± 76
k_{ex}^{A-B} 278K (s^{-1})	1489 ± 286	749 ± 430	629 ± 95
k_{ex}^{A-C} 298K (s^{-1})	466 ± 236	3184 ± 327	
k_{ex}^{A-C} 288K (s^{-1})	217 ± 191	2045 ± 210	
k_{ex}^{A-C} 278K (s^{-1})	101 ± 147	1900 ± 362	

HDAC8:TCSA (in D_2O)

	L1	L2,6	CAT
p(B) 298K (%)	17.1 ± 1.4	4.5 ± 1.5	1.4 ± 0.2
p(B) 288K (%)	7.7 ± 0.5	8.0 ± 3.2	2.6 ± 0.2
p(B) 278K (%)	2.8 ± 0.3	12.6 ± 5.8	5.0 ± 0.3
p(C) 298K (%)	3.2 ± 0.8	3.2 ± 1.0	
p(C) 288K (%)	8.4 ± 0.6	9.4 ± 2.6	
p(C) 278K (%)	20.5 ± 3.6	25.3 ± 6.6	
k_{ex}^{A-B} 298K (s^{-1})	1000 ± 55	1032 ± 332	3051 ± 351
k_{ex}^{A-B} 288K (s^{-1})	774 ± 56	432 ± 161	2644 ± 181
k_{ex}^{A-B} 278K (s^{-1})	627 ± 95	178 ± 77	2297 ± 186
k_{ex}^{B-C} 298K (s^{-1})	8318 ± 2265	2670 ± 906	
k_{ex}^{B-C} 288K (s^{-1})	2155 ± 414	1702 ± 551	
k_{ex}^{B-C} 278K (s^{-1})	1092 ± 243	1127 ± 346	

Cross validation: M274A-HDAC8 (in D_2O)

	L1	L2,6	CAT
p(B) 298K (%)	2.7 ± 4.6	0.25 ± 0.15	8.1 ± 1.4
p(B) 288K (%)	10.0 ± 8.2	0.6 ± 0.3	9.9 ± 1.3
p(B) 278K (%)	27.0 ± 13.0	1.7 ± 1.0	12.3 ± 1.3
p(C) 298K (%)	1.5 ± 0.3	2.7 ± 1.2	
p(C) 288K (%)	6.9 ± 1.5	4.1 ± 0.9	
p(C) 278K (%)	24.4 ± 10.0	6.3 ± 3.1	
k_{ex}^{A-B} 298K (s^{-1})	7852 ± 5172	3122 ± 3975	4191 ± 213
k_{ex}^{A-B} 288K (s^{-1})	4855 ± 1415	1942 ± 1166	1716 ± 78
k_{ex}^{A-B} 278K (s^{-1})	3694 ± 1885	1107 ± 806	662 ± 49
k_{ex}^{A-C} 298K (s^{-1})	436 ± 295	2565 ± 877	
k_{ex}^{A-C} 288K (s^{-1})	411 ± 174	871 ± 146	
k_{ex}^{A-C} 278K (s^{-1})	503 ± 275	278 ± 136	

Table S4. The 93 states used to simulate kinetic parameters of HDAC8 were defined as follows

State no.	L1 conformation	L2,6 conformation	CAT conformation	HDAC8 species	Internal dynamics
0	Bound (B_{L1}^{free})	Bound ($B_{L2,6}^{free}$)	A	E	Free HDAC8
1	Free (A_{L1}^{free})	Bound	A	E	Free HDAC8
2	Release (C_{L1}^{free})	Bound	A	E	Free HDAC8
3	Bound	Free ($A_{L2,6}^{free}$)	A	E	Free HDAC8
4	Free	Free	A	E	Free HDAC8
5	Release	Free	A	E	Free HDAC8
6	Bound	$C_{L2,6}^{free}$	A	E	Free HDAC8
7	Free	$C_{L2,6}^{free}$	A	E	Free HDAC8
8	Release	$C_{L2,6}^{free}$	A	E	Free HDAC8
9	Bound	Bound	B	E	Free HDAC8
10	Free	Bound	B	E	Free HDAC8
11	Release	Bound	B	E	Free HDAC8
12	Bound	Free	B	E	Free HDAC8
13	Free	Free	B	E	Free HDAC8
14	Release	Free	B	E	Free HDAC8
15	Bound	$C_{L2,6}^{free}$	B	E	Free HDAC8
16	Free	$C_{L2,6}^{free}$	B	E	Free HDAC8
17	Release	$C_{L2,6}^{free}$	B	E	Free HDAC8
18 [a)]	Bound (A_{L1}^{TCSA})	Bound ($A_{L2,6}^{TCSA}$)	A	ES	HDAC8:TCSA
19	Free (B_{L1}^{TCSA})	Bound	A	ES	HDAC8:TCSA
20	Release (C_{L1}^{TCSA})	Bound	A	ES	HDAC8:TCSA
21	Bound	Free ($B_{L2,6}^{TCSA}$)	A	ES	HDAC8:TCSA
22	Free	Free	A	ES	HDAC8:TCSA
23	Release	Free	A	ES	HDAC8:TCSA
24	Bound	$C_{L2,6}^{TCSA}$	A	ES	HDAC8:TCSA
25	Free	$C_{L2,6}^{TCSA}$	A	ES	HDAC8:TCSA
26	Release	$C_{L2,6}^{TCSA}$	A	ES	HDAC8:TCSA
27	Bound	Bound	B	ES	HDAC8:TCSA
28	Free	Bound	B	ES	HDAC8:TCSA
29	Release	Bound	B	ES	HDAC8:TCSA
30	Bound	Free	B	ES	HDAC8:TCSA
31	Free	Free	B	ES	HDAC8:TCSA
32	Release	Free	B	ES	HDAC8:TCSA
33	Bound	$C_{L2,6}^{TCSA}$	B	ES	HDAC8:TCSA
34	Free	$C_{L2,6}^{TCSA}$	B	ES	HDAC8:TCSA
35	Release	$C_{L2,6}^{TCSA}$	B	ES	HDAC8:TCSA
36 [a)]	Bound	Bound	A	$EP_{lys}P_{ace}$	HDAC8:TCSA
37	Free	Bound	A	$EP_{lys}P_{ace}$	HDAC8:TCSA
38	Release	Bound	A	$EP_{lys}P_{ace}$	HDAC8:TCSA
39	Bound	Free	A	$EP_{lys}P_{ace}$	HDAC8:TCSA
40	Free	Free	A	$EP_{lys}P_{ace}$	HDAC8:TCSA
41	Release	Free	A	$EP_{lys}P_{ace}$	HDAC8:TCSA
42	Bound	$C_{L2,6}^{TCSA}$	A	$EP_{lys}P_{ace}$	HDAC8:TCSA
43	Free	$C_{L2,6}^{TCSA}$	A	$EP_{lys}P_{ace}$	HDAC8:TCSA
44	Release	$C_{L2,6}^{TCSA}$	A	$EP_{lys}P_{ace}$	HDAC8:TCSA
45	Bound	Bound	B	$EP_{lys}P_{ace}$	HDAC8:TCSA

46	Free	Bound	B	$EP_{lys}P_{ace}$	HDAC8:TCSA
47	Release	Bound	B	$EP_{lys}P_{ace}$	HDAC8:TCSA
48	Bound	Free	B	$EP_{lys}P_{ace}$	HDAC8:TCSA
49	Free	Free	B	$EP_{lys}P_{ace}$	HDAC8:TCSA
50	Release	Free	B	$EP_{lys}P_{ace}$	HDAC8:TCSA
51	Bound	$C_{L2,6}^{TCSA}$	B	$EP_{lys}P_{ace}$	HDAC8:TCSA
52	Free	$C_{L2,6}^{TCSA}$	B	$EP_{lys}P_{ace}$	HDAC8:TCSA
53	Release	$C_{L2,6}^{TCSA}$	B	$EP_{lys}P_{ace}$	HDAC8:TCSA
54	Bound	Bound	A	EP_{ace}	Free HDAC8
55	Free	Bound	A	EP_{ace}	Free HDAC8
56	Release	Bound	A	EP_{ace}	Free HDAC8
57	Bound	Free	A	EP_{ace}	Free HDAC8
58	Free	Free	A	EP_{ace}	Free HDAC8
59	Release	Free	A	EP_{ace}	Free HDAC8
60	Bound	$C_{L2,6}^{free}$	A	EP_{ace}	Free HDAC8
61	Free	$C_{L2,6}^{free}$	A	EP_{ace}	Free HDAC8
62	Release	$C_{L2,6}^{free}$	A	EP_{ace}	Free HDAC8
63	Bound	Bound	B	EP_{ace}	Free HDAC8
64	Free	Bound	B	EP_{ace}	Free HDAC8
65	Release	Bound	B	EP_{ace}	Free HDAC8
66	Bound	Free	B	EP_{ace}	Free HDAC8
67	Free	Free	B	EP_{ace}	Free HDAC8
68	Release	Free	B	EP_{ace}	Free HDAC8
69	Bound	$C_{L2,6}^{free}$	B	EP_{ace}	Free HDAC8
70	Free	$C_{L2,6}^{free}$	B	EP_{ace}	Free HDAC8
71	Release	$C_{L2,6}^{free}$	B	EP_{ace}	Free HDAC8
72	Bound	Bound	A	EP_{lys}	HDAC8:TCSA
73	Free	Bound	A	EP_{lys}	HDAC8:TCSA
74	Release	Bound	A	EP_{lys}	HDAC8:TCSA
75	Bound	Free	A	EP_{lys}	HDAC8:TCSA
76	Free	Free	A	EP_{lys}	HDAC8:TCSA
77	Release	Free	A	EP_{lys}	HDAC8:TCSA
78	Bound	$C_{L2,6}^{TCSA}$	A	EP_{lys}	HDAC8:TCSA
79	Free	$C_{L2,6}^{TCSA}$	A	EP_{lys}	HDAC8:TCSA
80	Release	$C_{L2,6}^{TCSA}$	A	EP_{lys}	HDAC8:TCSA
81	Bound	Bound	B	EP_{lys}	HDAC8:TCSA
82	Free	Bound	B	EP_{lys}	HDAC8:TCSA
83	Release	Bound	B	EP_{lys}	HDAC8:TCSA
84	Bound	Free	B	EP_{lys}	HDAC8:TCSA
85	Free	Free	B	EP_{lys}	HDAC8:TCSA
86	Release	Free	B	EP_{lys}	HDAC8:TCSA
87	Bound	$C_{L2,6}^{TCSA}$	B	EP_{lys}	HDAC8:TCSA
88	Free	$C_{L2,6}^{TCSA}$	B	EP_{lys}	HDAC8:TCSA
89	Release	$C_{L2,6}^{TCSA}$	B	EP_{lys}	HDAC8:TCSA
90	Substrate, S *or* inhibitor, I				
91	Lysine product, P_{lys}				
92	Acetate product, P_{ace}				

a) Hydrolysis is described by a first-order reaction with rate k_H from state 18 to state 36. Binding of substrate, as well as dissociation of substrate and product is defined as described in Fig 4. For analysis of non-hydrolysable inhibitor-binding only states 0-35 and 90 are considered with k_H = 0 s^{-1}.

Table S5. Experimental parameters and top kinetic models derived

Panel a: Experimental parameters used in least-squares analysis

Protein	k_{cat} (s^{-1})	K_M (M)	k_{cat}/K_M ($s^{-1}M^{-1}$)	K_d (µM)	$k_{off,TCSA,macro}$ (s^{-1})
Wild-type in H_2O	0.90 ± 0.09	0.023 ± 0.002	39.0 ± 4.0	5.3 ± 0.3	n/a
Wild-type in D_2O	n/a	n/a	6.3 ± 0.8	5.0 ± 2.0	0.13 ± 0.10
L179A in H_2O	n/a	n/a	2.7 ± 1.0	110 ± 11	0.19 ± 0.20
**Cross-validation**					
M274A in H_2O	n/a	n/a	1.2 ± 1.3	116 ± 12	1.19 ± 0.15

Panel b: Top kinetic models with p-value larger than 0.05

Model	Substrate association and dissociation			Substrate and lysine product dissociation			χ^2	χ^2_{red}	p-value[a)]	χ^2_{cross}[b)]
	CAT	L1	L2,6	CAT	L1	L2,6				
1[c)]	B	Bound	C_{L6}	B	Bound	Free	4.43	1.11	-	5.5
2[d)]	B	Bound	Free	B	Bound	C_{L6}	4.56	1.14	0.717	5.4
3	A&B	C_{L1}	C_{L6}	B	Bound	Free	6.23	1.56	0.180	62.1
4	A	C_{L1}	C_{L6}	B	Bound	Free	6.31	1.58	0.171	63.0
5	B	Free	Bound	B	Bound	C_{L6}	6.41	1.60	0.160	238.4
6	A	Bound	C_{L6}	B	all	Free	6.67	1.67	0.135	123.5
7	A&B	Bound	C_{L6}	B	all	Free	6.68	1.67	0.134	123.1
8	B	Bound	Free	A&B	C_{L1}	C_{L6}	6.74	1.69	0.129	64.2
9	B	Free	Bound	A	Bound	C_{L6}	6.77	1.69	0.127	305.8
10	A	Bound	C_{L6}	B	Free	Bound	6.78	1.69	0.126	305.9
11	B	C_{L1}	Free	A	C_{L1}	C_{L6}	6.78	1.70	0.125	64.7
12	B	Free	Bound	A&B	Bound	C_{L6}	6.79	1.70	0.124	293.9
13	A&B	Bound	C_{L6}	B	Free	Bound	6.80	1.70	0.124	294.1
14	B	Bound	Free	A	C_{L1}	C_{L6}	6.82	1.71	0.122	65.1
15	B	C_{L1}	Free	A&B	C_{L1}	C_{L6}	6.90	1.73	0.116	63.9
16	B	all	Free	A	Bound	C_{L6}	6.91	1.73	0.116	124.8
17	B	all	Free	A&B	Bound	C_{L6}	6.93	1.73	0.114	124.4
18	B	Free	Free	A	Free	C_{L6}	6.95	1.74	0.112	64.4
19	B	Free	Free	A	Bound	C_{L6}	7.02	1.75	0.108	127.0
20	B	Free	Free	A&B	Bound	C_{L6}	7.03	1.76	0.107	126.6
21	B	Free	Free	A&B	Free	C_{L6}	7.08	1.77	0.104	63.6
22	B	Free	all	A&B	C_{L1}	C_{L6}	7.11	1.78	0.101	127.5
23	B	Free	all	A	C_{L1}	C_{L6}	7.12	1.78	0.101	127.7
24	B	all	Free	A	Free	C_{L6}	7.15	1.79	0.099	63.4
25	A	Bound	C_{L6}	B	Bound	Free	7.16	1.79	0.099	64.7
26	B	Free	Bound	A	C_{L1}	C_{L6}	7.17	1.79	0.098	255.7
27	A&B	Bound	C_{L6}	B	Bound	Free	7.29	1.82	0.091	64.0
28	B	Free	Bound	A&B	C_{L1}	C_{L6}	7.29	1.82	0.091	249.9
29	B	all	Free	A&B	Free	C_{L6}	7.30	1.82	0.090	62.8
30	A&B	C_{L1}	C_{L6}	A&B	C_{L1}	Free	7.39	1.85	0.085	38.1
31	A	C_{L1}	C_{L6}	A	C_{L1}	Free	7.39	1.85	0.085	37.1
32	A&B	C_{L1}	C_{L6}	A	C_{L1}	Free	7.40	1.85	0.085	36.8

33	A	C_{L1}	C_{L6}	A&B	C_{L1}	Free	7.41	1.85	0.084	38.5
34	B	Bound	Free	A	Bound	C_{L6}	7.49	1.87	0.081	65.3
35	A	C_{L1}	Free	A	C_{L1}	C_{L6}	7.49	1.87	0.080	38.7
36	A&B	C_{L1}	Free	A&B	C_{L1}	C_{L6}	7.49	1.87	0.080	39.7
37	A&B	C_{L1}	Free	A	C_{L1}	C_{L6}	7.49	1.87	0.080	40.0
38	A	C_{L1}	Free	A&B	C_{L1}	C_{L6}	7.50	1.87	0.080	38.4
39	B	Bound	Free	A&B	Bound	C_{L6}	7.62	1.90	0.074	64.6
40	A	Bound	C_{L6}	B	all	Bound	7.70	1.93	0.071	284.1
41	B	all	Bound	A	Bound	C_{L6}	7.70	1.93	0.070	283.0
42	B	Bound	C_{L6}	B	Free	Bound	7.73	1.93	0.069	238.0
43	A	Bound	Free	A&B	Bound	C_{L6}	7.81	1.95	0.066	13.7
44	A	Bound	Free	A	Bound	C_{L6}	7.82	1.95	0.066	13.9
45	A&B	Bound	Free	A	Bound	C_{L6}	7.82	1.95	0.066	15.1
46	A&B	Bound	Free	A&B	Bound	C_{L6}	7.82	1.96	0.066	14.8
47	A&B	Bound	C_{L6}	A	Bound	Free	7.82	1.96	0.066	13.7
48	A&B	Bound	C_{L6}	A&B	Bound	Free	7.83	1.96	0.065	14.9
49	A	Bound	C_{L6}	A	Bound	Free	7.83	1.96	0.065	13.9
50	A	Bound	C_{L6}	A&B	Bound	Free	7.83	1.96	0.065	15.1
51	A&B	Bound	C_{L6}	B	all	Bound	7.87	1.97	0.064	271.0
52	B	all	Bound	A&B	Bound	C_{L6}	7.90	1.97	0.063	270.8
53	A&B	C_{L1}	C_{L6}	B	Free	all	7.97	1.99	0.060	113.7
54	A	C_{L1}	C_{L6}	B	Free	all	7.97	1.99	0.060	114.0
55	A	C_{L1}	C_{L6}	B	Free	Bound	8.06	2.01	0.057	253.9
56	A	Bound	C_{L6}	B	Free	all	8.12	2.03	0.055	118.2
57	A&B	C_{L1}	C_{L6}	B	all	Free	8.14	2.04	0.054	110.6
58	A&B	Bound	C_{L6}	B	Free	all	8.15	2.04	0.054	117.8
59	A	C_{L1}	C_{L6}	B	all	Free	8.16	2.04	0.053	110.8
60	A	Free	Free	A	Free	C_{L6}	8.17	2.04	0.053	21.7
61	B	Free	all	A	Bound	C_{L6}	8.18	2.04	0.053	118.9
62	A	Free	Free	A&B	Free	C_{L6}	8.18	2.05	0.053	21.2
63	A&B	Free	Free	A	Free	C_{L6}	8.18	2.05	0.053	23.3
64	A&B	Free	Free	A&B	Free	C_{L6}	8.19	2.05	0.053	22.9
65	A&B	C_{L1}	C_{L6}	B	Free	Bound	8.19	2.05	0.052	248.1
66	B	Free	all	A&B	Bound	C_{L6}	8.21	2.05	0.052	118.4
67	A	Free	C_{L6}	A	Free	Free	8.22	2.05	0.052	21.5
68	A&B	Free	C_{L6}	A	Free	Free	8.22	2.06	0.052	21.0
69	A	Free	C_{L6}	A&B	Free	Free	8.23	2.06	0.051	23.1
70	A&B	Free	C_{L6}	A&B	Free	Free	8.23	2.06	0.051	22.7
	**Conformational selection model**									
349	A	Bound	Bound	A	Bound	Bound	23.27	5.82	1.43×10^{-5}	210.0

[a] p-value compared with Model 1, calculated using the model number as a discrete parameter, see Material and Methods. The states are as follows: 'A' and 'B' are the ground-state and low-populated state within the catalytic region, respectively. 'Bound' is the bound-like state, that is, $\mathrm{A}_{\mathrm{L1}}^{\mathrm{TCSA}}$ and $\mathrm{A}_{\mathrm{L2,6}}^{\mathrm{TCSA}}$ in the bound form and $\mathrm{B}_{\mathrm{L1}}^{\mathrm{free}}$ and $\mathrm{B}_{\mathrm{L2,6}}^{\mathrm{free}}$ in the free form, 'Free' are the $\mathrm{B}_{\mathrm{L1}}^{\mathrm{TCSA}}$ and $\mathrm{B}_{\mathrm{L2,6}}^{\mathrm{TCSA}}$ states of the bound form and the $\mathrm{A}_{\mathrm{L1}}^{\mathrm{free}}$ and $\mathrm{A}_{\mathrm{L2,6}}^{\mathrm{free}}$ states of the free enzyme. A notation of 'all' means that all states, A, B, and C, are participating in association and/or dissociation.

[b] Cross-validation χ^2 calculated from the obtained k_{cat}/K_M, K_d, and $k_{off,In,macro}$ for the M274A-HDAC8 mutant.

[c] See Fig 4b for a detailed description of this model. Optimized parameters are k_{on} = 4.0 ± 1.3 μM^{-1} s^{-1}, k_{off} = 5.1×10^5 ± 1.7×10^5 s^{-1} , $k_{off,I}$ = 124 ± 40 s^{-1}, k_H (H_2O) = 2.04 ± 0.16 s^{-1}.

[d] Optimized parameters are k_{on} = 4.0 ± 1.3 μM^{-1} s^{-1}, k_{off} = 6.9×10^5 ± 2.3×10^5 s^{-1}, $k_{off,I}$ = 124 ± 40 s^{-1}, k_H(H_2O) = 2.9 ± 0.3 s^{-1}.

SI References

1. Q. Sun, The Raman OH stretching bands of liquid water. *Vib. Spectrosc.* **51**, 213–217 (2009).
2. L. Pogliani, The detailed balance principle in matrix kinetics. *React. Kinet. Catal. Lett.* **64**, 9–14 (1998).
3. J. L. Markley, *et al.*, BioMagResBank (BMRB) as a partner in the Worldwide Protein Data Bank (wwPDB): new policies affecting biomolecular NMR depositions. *J. Biomol. NMR* **40**, 153–155 (2008).
